# Companion planting with French marigolds protects tomato plants from glasshouse whiteflies through the emission of airborne limonene

**DOI:** 10.1371/journal.pone.0213071

**Published:** 2019-03-01

**Authors:** Niall J. A. Conboy, Thomas McDaniel, Adam Ormerod, David George, Angharad M. R. Gatehouse, Ellie Wharton, Paul Donohoe, Rhiannon Curtis, Colin R. Tosh

**Affiliations:** 1 School of Natural and Environmental Sciences, Newcastle University, Newcastle-upon-Tyne, United Kingdom; 2 Stockbridge Technology Centre, North Yorkshire, United Kingdom; 3 Northumbria University, Faculty of Engineering and Environment, Newcastle-upon-Tyne, United Kingdom; Institut Sophia Agrobiotech, FRANCE

## Abstract

Horticulturalists and gardeners in temperate regions often claim that planting marigolds next to tomato plants protects the tomatoes from the glasshouse whitefly (*Trialeurodes vaporariorum* Westwood). If shown to hold true, this technique could be used in larger-scale tomato production, protecting the crop and helping to introduce greater plant diversity into these agro-ecosystems. Here we present two large-scale glasshouse trials corresponding to the two main ways growers are likely to use marigolds to control whiteflies. In the first, marigolds are grown next to tomato throughout the growing period and we quantify whitefly population growth from the seedling stage over a 48 day infestation period. Here we show that association with marigolds significantly slows whitefly population development. Introducing additional whitefly-attractive ‘pull’ plants around the perimeter of plots has little effect, but reducing the proportion of marigolds and introducing other non-hosts of whiteflies (basil, nasturtium and Chinese cabbage) also reduces whitefly populations on tomato. The second experiment assesses the efficacy of marigolds when used as an ‘emergency’ measure. Here we allow whitefly populations to build to a high density on unprotected tomatoes then introduce marigolds and assess whitefly population over a further period. Following laboratory work showing limonene to be a major chemical component of French marigolds and a negative behaviour response of whiteflies to this compound, limonene dispensers are added as an additional treatment to this experiment. “Emergency” marigold companion planting yielded minimal reductions in whitefly performance, but the use of limonene dispensers was more effective. Our work indicates that companion planting short vine tomatoes with French marigolds throughout the growing season will slow development of whitefly populations. Introducing marigolds to unprotected tomatoes after significant whitefly build-up will be less effective. The use of limonene dispensers placed near to tomato plants also shows promise. It is argued that this work supports the possibility of the development of a mixture of tomato companion plants that infer ‘associational resistance’ against many major invertebrate pests of tomato. Such a mixture, if comprising edible or ornamental plants, would be economically viable, would reduce the need for additional chemical and biological control, and, if used outdoors, would generate plant-diverse agro-ecosystems that are better able to harbour invertebrate wildlife.

## Introduction

Tomatoes (*Solanum lycopersicum* L.) are the second most important edible horticultural crop by production in developed nations [1]. Under glass, pest infestations are dominated by the common glasshouse whitefly (*Trialeurodes vaporariorum*) [2] which is known to cause severe yield losses to an array of crops through transmission of a number of plant viruses [3, 4]. Direct feeding from both adults and larvae results in honeydew secretion which reduces the photosynthetic capacity of the plant and renders fruit unmarketable [5]. Biocontrol is widely used to manage *T*. *vaporariorum* infestations but limitations such as delayed efficacy [6] and hyperparasitism [7] can lead to failure. Where biocontrol fails, there is still reliance on chemical control for tomato production and excessive use of chemical sprays has resulted in resistant *T*. *vaporariorum* genotypes, phytotoxicity and pesticide residue problems [8–10]. As with most contemporary cropping systems, tomatoes are typically produced in monoculture, thus rendering crop areas of limited value to wildlife and largely devoid of “ecosystem services” [11]. Any alternative methods of whitefly control that can reduce pesticide use and introduce greater animal and plant diversity into agricultural and horticultural systems should therefore be welcomed. The insistence of temperate region gardeners that planting marigolds next to tomatoes protects the tomato crop from whiteflies therefore merits further investigation. Companion planting is a well-researched (and implemented) pest control strategy and is believed to be founded on ‘associational resistance’ [12, 13]. Both French (*Tagetes patula* L.) and English marigold (*Calendula officinalis*) have been used as effective companion plants in a number of other pest/crop scenarios. They have been shown to reduce pest populations either directly through repellent volatile chemistry [14] or indirectly through promoting beneficial arthropod populations [15–18]. Despite this, no research appears to be available that quantifies the potential for marigolds to control whiteflies on tomatoes.

Here, large-scale glasshouse trials in the United Kingdom are described investigating the potential of intercropping tomato plants with marigold and other plant species to repel the glasshouse whitefly, *T*. *vaporariorum*. One set of experiments investigated the effect of intercropping with other plant species for the duration of the tomato growth period. The following year, another experiment investigated the effect of introducing intercropped treatments into a tomato crop grown alone for the majority of the growing season, when a high density whitefly population had developed. This represents an “emergency” situation which horticulturists may experience if no previous control methods have been in place. These studies aimed to investigate: 1) Whether propagation of French marigolds (*Tagetes patula* L.) amongst tomato plants from the start of the growing period protects tomatoes from whitefly infestation by ‘pushing’ them from the tomato crop; 2) If supplementing marigolds over the whole growth period with other non-host species less preferred by whitefly, to ‘push’ whiteflies from tomatoes, increases the marigold protective effect; 3) Whether this protection may be enhanced by positioning preferred ‘pull’ whitefly host plants around the edge of the ‘push’ non-hosts [19], 4) Using laboratory studies, what is the main chemical component of marigold airborne volatiles and do they have behavioural activity against whiteflies? 5) Whether the introduction of marigolds and limonene (identified from 4 above) dispensers can protect tomatoes in an “emergency” situation where there is a heavy whitefly infestation on the tomato crop. The investigation into whether increased diversity achieves greater levels of whitefly control was included because, from an ecosystem health perspective, plant diversity is desirable, but also because plant diversity is known to be a fundamental determinant of invertebrate abundance [20, 21]. Plant diversity in agroecosystems is also important for natural enemies and marigolds have been found to increase populations of beneficial arthropods [17, 18]. In contrast, the current work was conducted in a closed glasshouse with no biocontrol present and little opportunity for natural enemies to enter. No natural enemies or parasitized pupae were observed in either glasshouse experiment and whilst they may have still been present in very low numbers, their effect on plant-whitefly interactions in both trials can be assumed to be minimal. Therefore, any effects on whitefly performance were most likely due to direct effects between the introduced non-host plants and whitefly. Non-host plants can act as a repellent to pests [14], make host plants harder to find (disruptive crop hypothesis) [22] and presence of multiple non-hosts have been found to induce ‘restlessness’ or ‘confused’ behaviour in the whitefly species *Bemisia tabaci* [23]. The current work aimed to investigate if these effects from non-host companion planting translate to lower rates of whitefly population development on tomato.

## Results and discussion

The first large scale glasshouse study sought to establish a scientific basis for the propagation of French marigolds amongst tomato plants from the start of the growing season to protect tomatoes from whitefly infestation. This work aimed to identify whether this practice achieved significant control of the important glasshouse pest *T*. *vaporariorum*, and whether this control could be enhanced by other aversive, non-host plants. A second treatment also sought to quantify whether this ‘push’ effect could be combined with the ‘pull’ of attractive host plants to increase whitefly control. French marigold was found to be an effective companion plant and subsequent headspace analysis followed by laboratory bioassays confirmed the volatile limonene to be the probable mechanistic basis of the repellent properties this plant possesses. In a second glasshouse trial, the potential for marigold plants and limonene dispensers to control a heavy infestation of whiteflies on tomato was assessed.

### Glasshouse trial 1: “Push”, “Push-Pull” and “High Diversity” control strategies applied at the beginning of the infestation period

**Fig 1:**
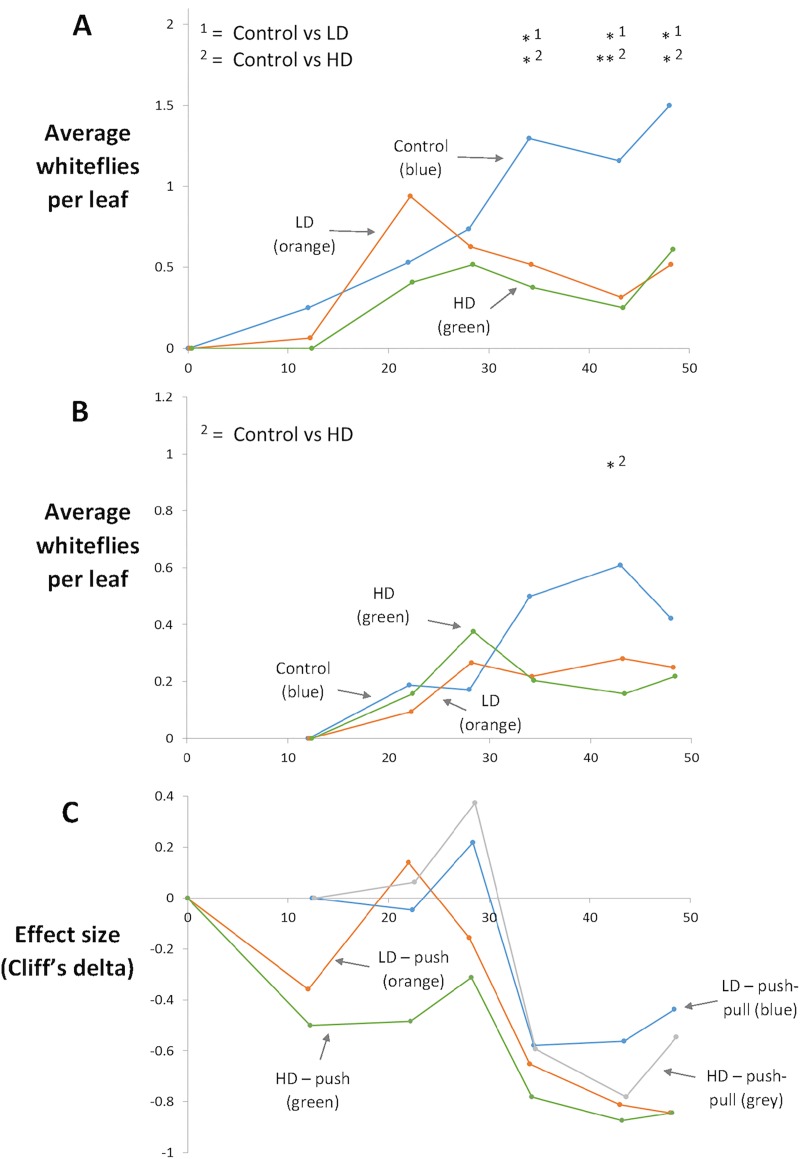
Population development of the whitefly, *T*. *vaporariorum* on tomato in the glasshouse, with all whitefly life stages (adult, eggs and nymphs) contributing to the average number of whitefly/leaf (*n =* 8). Day 0 is 10th August 2016 and the ‘push-pull’ was started 12 days after the “push” experiment. Whitefly abundance data from Fig 1A and 1B was (log +1) transformed and analysed over time with repeated measures ANOVA’s and Bonferroni corrected post-hoc comparisons, in which the *p* values were multiplied by the total number of comparisons (*n =* 3). Significant observations between treatments at individual sampling points are annotated onto the graphs with asterisks, “*” indicates a *p* value of <0.05, “**” *p* <0.001. All other *p* values were non-significant at *α* = 0.05. Fig 1A Shows the “push” experiment in which repellent plants such as marigold (LD) and marigold plus non-hosts (HD) are intercropped amongst tomato plants. There was a significant effect of the treatment (rm ANOVA *F*_(2,126)_ = 18.85, *p <* 0.001) and treatment *x* time (rm ANOVA *F*_(10,126)_ = 2.14, *p =* 0.025) across the sampling period. Fig 1B Shows the ‘push-pull’ experiment which is the same as the ‘push’ experiment but additionally a single (LD) and several (HD) host plant species surround the repellent hosts and tomato. There was a significant effect of treatment (*F*_(2,105)_ = 3.73, *p =* 0.027) but no significant effect of treatment *x* time (*F*_(8,105)_ = 1.49, *p =* 0.166) following repeated measures ANOVA’s. Post-hoc comparisons from Fig 1A showed there were significantly less whiteflies in LD and HD plots at day 34 (LD, *t =* 2.89, *df =* 126, *p =* 0.013. HD, *t =* 3.61, *df =* 126, *p =* 0.001), day 43 (LD, *t =* 3.59, *df =* 126, *p =* 0.001. HD, *t =* 3.92, *df =* 126, *p <* 0.001) and day 48 (LD, *t =* 3.66, *df =* 126, *p =* 0.001. HD, *t =* 3.29, *df =* 126, *p =* 0.003). For Fig 1B, there were significantly less whiteflies on the HD treatment at day 43 (*t =* 3.018, *df =* 105, *p =* 0.009) but not on the LD treatment (*t =* 2.20, *df =* 105, *p =* 0.088). Fig 1C shows the data in Fig 1A and 1B expressed as effect size (relative to control). The Cliff’s *d* measure is used as this is suitable for non-normal data of the type observed in experiments. Values of 1 or -1 (the sign shows the direction of effects relative to control) indicate complete non-overlap between the groups under consideration and a value of 0 indicates complete overlap. Ninety five percent confidence intervals have been calculated and are available in the supporting information (S1 Dataset), but to aid visualisation they have been removed from the figure.

Fig 1 shows levels of whitefly infestation on the tomato crop through time. In the ‘push’ experiment (Fig 1A) whitefly numbers on the control (tomato plants only), low diversity (LD, tomato intercropped with marigold) and high diversity (HD, tomato intercropped with marigold and other *T*. *vaporariorum* non-hosts) treatments began to diverge after 34 days. There was a significant effect of treatment (Control, LD and HD)(repeated measures (rm) ANOVA *F*_(2,126)_ = 18.85, *p <* 0.001) and treatment *x* time (each sampling point, n = 6) (rm ANOVA *F*_(10,126)_ = 2.14, *p =* 0.025) in the “push” experiment (Fig 1A). There was no difference in whitefly abundance between the treatments until day 34 where there were significantly less whiteflies on tomatoes in LD (*t =* 2.89, *df =* 126, *p =* 0.013) and HD (*t =* 3.61, *df =* 126, *p =* 0.001) plots. This trend continued until the final sampling point at day 48. Statistical comparisons were made between whitefly population size in the HD and LD treatments but there was no significant differences observed. This indicates that increasing the diversity of non-hosts does not improve the repellent effect (conversely, and importantly, it does not reduce the potency of the marigold-only effect). In the ‘push-pull’ experiment (Fig 1B), was a significant effect of treatment (rm ANOVA *F*_(2,105)_ = 3.73, *p =* 0.027) but no significant interaction between treatment *x* time (rm ANOVA *F*_(8,105)_ = 1.49, *p =* 0.166). Average whitefly numbers on tomato were fewer on LD and HD treatments relative to the control after around 20 days and there were significantly fewer whiteflies in the HD treatment at day 43 (*t =* 3.018, *df =* 105, *p =* 0.009). It is important to note that relatively low levels of whitefly numbers were present in the greenhouse. Fig 1C shows that while the ‘push-pull’ experiment reached its maximum effect sooner than the ‘push’ experiment, it did not produce a greater maximum effect. Additionally, there was no significant difference in the effect of LD and HD treatments within experiments. It is, therefore, doubtful that growers could be persuaded to make the extra effort to propagate ‘pull’ plants on the basis of these results. Fig 1C should be viewed with caution as experiments were terminated at the end of the commercial growing season and effects may have diverged further (or disappeared) if planting had begun earlier.

While some studies have shown a positive relationship between plant species richness and insect abundance, this is thought to be related to plant productivity [20]. The artificial way in which glasshouse plants are propagated, i.e. with physically separated roots, as well as the short term nature of the experiments, may preclude such effects. In contrast, it was hypothesised that increased plant diversity (the HD treatment) would depress whitefly population growth on tomato, because a previous study showed increased plant diversity causes whiteflies to become ‘restless’ and move around diversified cultures more than simple, single species ones [23], presumably reducing time available for reproductive output. However, this hypothesis was not upheld, and increased plant diversity appeared relatively neutral with regard to whitefly performance on tomato in the current work. While these effects produced by marigold and other non-hosts are exciting, they are of even more relevance if the planting regime does not attract other pests to tomato. The only other pest to infest the experiment in significant numbers was the onion thrips (*Thrips tabaci*) there were no significant differences in larvae and adult abundance between treatments in either the “push” or “push-pull” experiment (S1 Fig). Other pests were found during the trial, but in much lower numbers, so were not included in the analysis. Plants were selected for the experiment specifically with glasshouse whiteflies in mind and it was not predicted that they would also repel other pests; therefore the lack of any significant effect on other pest insects is encouraging for the future development of this method. Total above ground fresh weight and fruit production was assessed at the end of the experimental period for every tomato plant in each of the three treatments, although no significant differences were observed between any of the treatments (S2 Fig).

#### Mechanistic basis of French marigold intercropping

Whilst repellent volatile chemistry is thought to be a major mode of action of companion plants [14], in the glasshouse experiment described above plants were kept in communal drip trays which would have allowed the sharing of root exudates between species. This phenomenon has been shown to trigger complex behavioural changes in *Deschampsia caespitosa* and *Arabidopsis thaliana* plants [24, 25]. Therefore a series of bioassays were conducted to see if this cross-species sharing of exudates could have influenced whitefly population development on tomato. No change in *T*. *vaporariorum* preference or performance (oviposition) was observed when tomato seedlings were previously exposed to marigold root exudates (S3 Fig). This indicates that repellent volatile organic compounds from marigold are the probable cause of the reduction in whitefly performance on tomato intercropped with marigold (Fig 1). Following this finding, headspace analysis of marigold seedlings was conducted in order to identify the repellent volatiles in operation. Limonene, a compound known to have insecticidal activities against a number of arthropods [26] including the Silverleaf whitefly (*B*. *tabaci*) [27–29], was found to be the most abundant volatile released from both flowers (24.01% of volatile output) and leaf tissue (21.04%) of marigold seedlings (S4 Fig).

**Fig 2:**
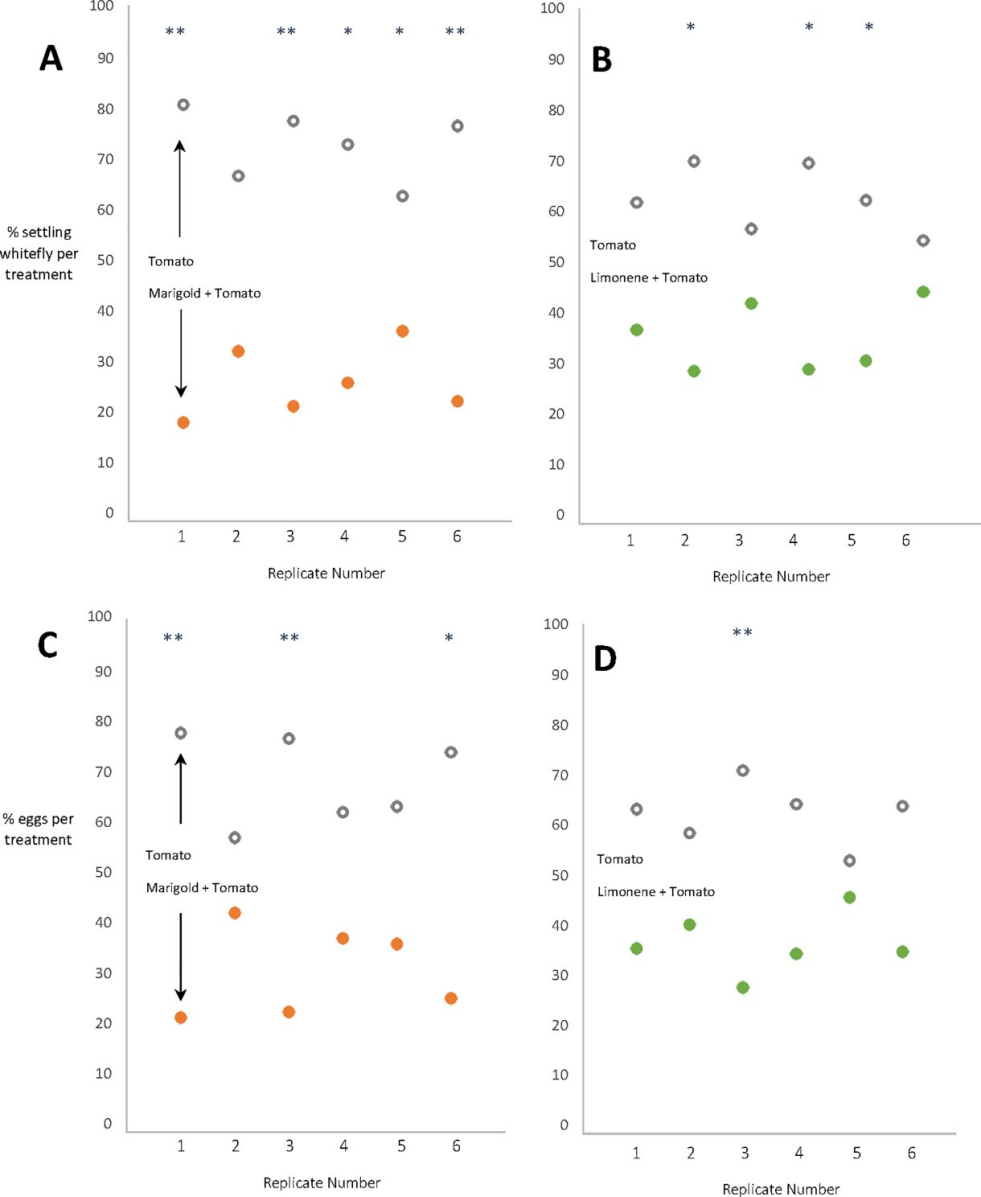
The percentage of whiteflies on tomato (*n* = 200) when given the choice between four un-accompanied “Elegance” tomato seedlings or four “Elegance” seedlings intercropped with either flowering marigold plants (Fig 2A) or limonene dispensers (Fig 2B). Whitefly eggs were also recorded for each replicate (Fig 2C and 2D). Values were transformed to percentages and the Pearson’s chi-squared test was used to test differences in settling and oviposition behaviour. Significant differences are annotated onto the graph for each treatment with the following format; “*” indicates a *p* value of <0.05, “**” *p* <0.001. Comparisons between individual replicates and controls for all four graphs (Fig 2A, 2B, 2C and 2D) are as follows; *df* = 1 for all reps, Fig 2A, rep 1 *X* ^2^ = 21.26, *p* = <0.000; rep 2 *X* ^2^ = 3.43, *p* = 0.064; rep 3 *X* ^2^ = 17.01, *p* = <0.000; rep 4 *X* ^2^ = 11.17, *p* = 0.001; rep 5 *X* ^2^ = 5.95, *p* = 0.015; rep 6 *X* ^2^ = 15.72, *p* = <0.000. Fig 2B, rep 1 *X* ^2^ = 2.92, *p* = 0.087; rep 2 *X* ^2^ = 8.33, *p* = 0.004; rep 3 *X* ^2^ = 0.985, *p* = 0.321; rep 4 *X* ^2^ = 8.33, *p* = 0.004; rep 5 *X* ^2^ = 5.32, *p* = 0.021; rep 6 *X* ^2^ = 0.501, *p* = 0.479. Fig 2C, rep 1 *X* ^2^ = 13.82, *p* = <0.000; rep 2 *X* ^2^ = 0.323, *p* = 0.570; rep 3 *X* ^2^ = 12.65, *p* = <0.000; rep 4 *X* ^2^ = 1.65, *p* = 0.198; rep 5 *X* ^2^ = 2.05, *p* = 0.152; rep 6 *X* ^2^ = 9.51, *p* = 0.002. Fig 2D, rep 1 *X* ^2^ = 2.31, *p* = 0.128; rep 2 *X* ^2^ = 0.731, *p* = 0.398; rep 3 *X* ^2^ = 6.876, *p* = 0.009; rep 4 *X* ^2^ = 2.97, *p* = 0.084; rep 5 *X* ^2^ = 0.02, *p* = 0.887; rep 6 *X* ^2^ = 2.49, *p* = 0.114. On average over the 6 replicates, 21.93% of whiteflies settled on tomato intercropped with marigold and 36.12% whiteflies settled on tomato intercropped with limonene dispensers. 31.44% of eggs were laid on marigold intercropped tomato and 37.24% were laid on limonene intercropped tomato. Average distribution (*n* = 6) of whiteflies in limonene and marigold treatments were compared to ascertain whether marigold control was significantly more effective at repelling whitefly, however this was not the case (*X* ^2^ = 2.016, *p* = 0.156, *df* = 1).

Whilst controlling insect pests by increasing plant diversity in agro-ecosystems would have greater environmental benefit, the development of “low-risk” plant-based crop protection products, with favourable environmental credentials is still favourable to the use of synthetic chemical sprays. Being based on naturally occurring chemistry, such products are generally considered to offer a more sustainable alternative to conventional synthetic chemistry, particularly when considering their strong potential use in IPM programmes. Whilst issues such as phytotoxicity can arise from over-use of repellent volatile sprays, volatile dispensers alleviate this issue by releasing the compounds of interest more slowly, and this method of delivery was actually found to be more effective at controlling whitefly settling [28]. Additionally, volatile dispensers take up less production space in the glasshouse and do not require the upkeep/husbandry costs which could be associated with companion plants. Considering this, the use of repellent volatile dispensers may be a more attractive alternative for some horticulturalists. We therefore developed limonene dispensers similar to that used by Du, Han [28] in an attempt to “push” whitefly from the target crop in a similar manner to that observed with marigold intercropping. In laboratory assays, we found that the limonene dispensers repelled whitefly from tomato and to only a slightly lesser extent than marigold seedlings (Fig 2), with 36.12% of whitefly choosing limonene intercropped tomato and 26.93% choosing marigold intercropped tomato (comparison of distribution between both treatments, *X* ^2^ = 2.016, *p* = 0.156, *df* = 1). Subsequent 4-way olfactometry experiments presented odours to whitefly from marigold flowers, marigold leaf tissue and the limonene dispensers. All of these treatments showed a significant level of repellence to *T*. *vaporariorum* (S5 Fig). Marigold flowers were found to release approximately double the amount of limonene than that of marigold leaf tissue per gram of fresh weight (S4 Fig) with the level of repellence observed being proportional to the respective tissue area (S5 Fig). These experiments confirmed the effectiveness of limonene as a repellent to *T*. *vaporariorum* and led to limonene dispensers being used along with marigold plants in the following glasshouse trial.

From the glasshouse trial and subsequent laboratory experiments we conclude that the reduced whitefly populations observed on marigold intercropped tomato was due to repellent volatile chemistry. Other marigold species have been shown to be repellent to tobacco whitefly (*Bemisia tabaci*) [16], green peach aphid (*Myzus persicae* Sulzer) [14], cabbage aphid (*Brevicoryne brassicae* L.), flea beatles (*Phyllotreta*), small and large white butterly (*Piers rapae* L., *Piers brassicae* L.) and the diamondback moth (*Plutella xylostella* L.) [15].

### Glasshouse trial 2: “Emergency” Intercropping to protect tomatoes from an established heavy whitefly infestation

Having shown that marigolds repel whiteflies from tomatoes during the early stages of plant growth at relatively low whitefly density (Fig 1), and that limonene is a major volatile component of marigolds (S4 Fig) and repellent to whiteflies (Fig 2), a further treatment with the limonene dispensers was tested; here they were introduced to heavily infested tomato plots in the same way as marigold plants. Heavy infestations are likely to occur if no protective measures are deployed against whiteflies in tomato growing facilities and home gardens. At this stage, even if more environmentally sound measures such as biocontrol are utilised, they are notoriously slow to take effect [6] and introduction of repellent plants or volatile dispensers could have a more immediate impact on whitefly infestations. If found to be capable of reducing the impact of a heavy whitefly infestation, then marigolds and/or limonene could be used instead of a chemical pesticide application, reducing the environmental impact of synthetic, conventional chemical whitefly control.

**Fig 3:**
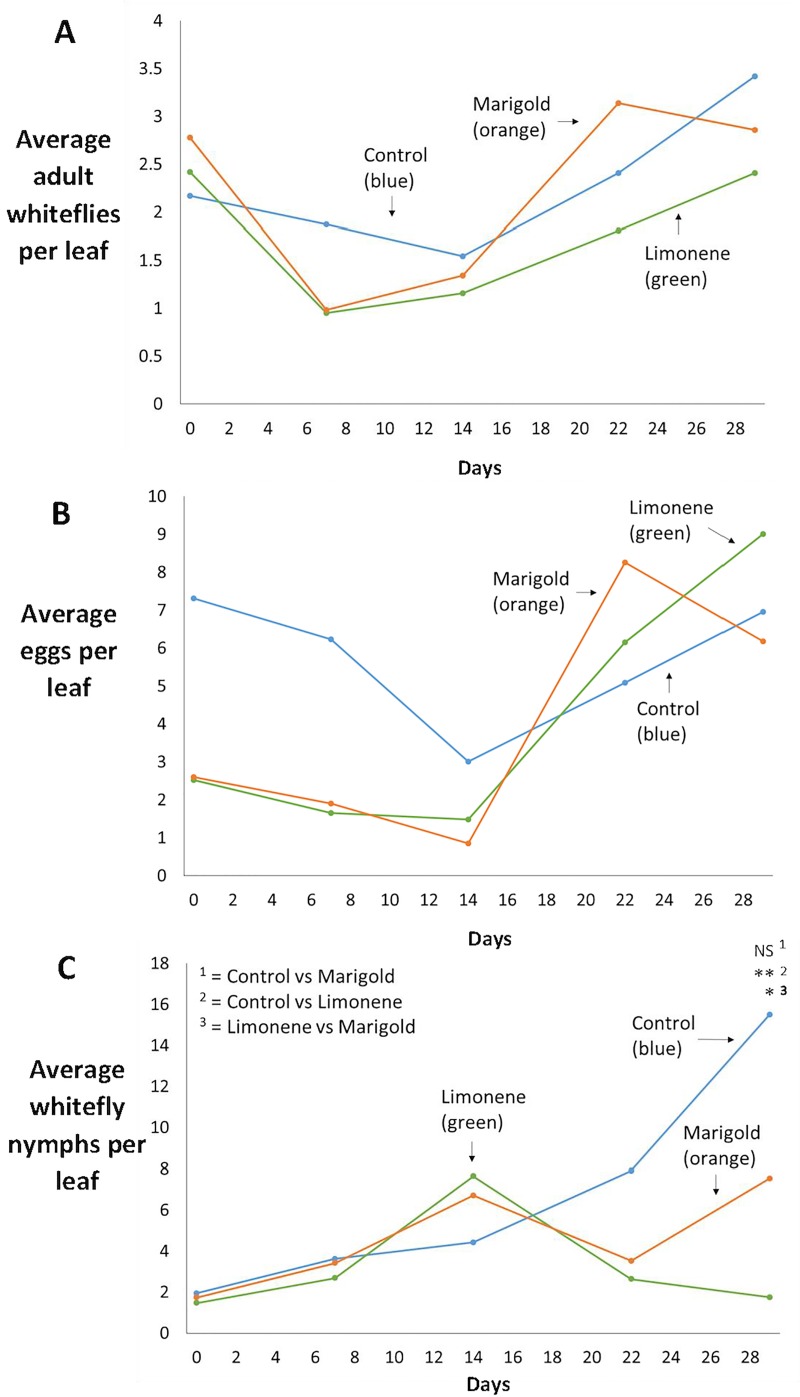
Whitefly population development in the second glasshouse trial with marigolds and limonene “emergency” intercropping treatments to control an established whitefly population (n = 8). Treatments comprised intercropping eight heavily whitefly-infested tomato plants with eight further tomato plants (control), eight French marigold plants (marigold treatment), or eight limonene dispensers (limonene treatment). The first data point in Fig 3A, 3B and 3C represents whitefly abundance (adults/eggs/nymphs) on the focal tomato plants immediately before treatment plants were introduced. ‘*’ indicates a *p* value of <0.05, ‘**’ indicates a *p* value of <0.001, NS = not significant. Fig 3A displays the average number of adult whitefly settled on each focal tomato plant in the three treatments over the course of the experiment (*n* = 8). No significant effect on settling adult whiteflies was observed between treatment (rm ANOVA *F*_(2,105)_ = 1.71, *p =* 0.185) and treatment *x* time (rm ANOVA *F*_(8.105)_ = 0.896, *p =* 0.522) across the sampling period. Fig 3B displays the average number of eggs laid on each focal tomato plant over the course of the experiment (*n* = 8). No significant effect on whitefly egg abundance was observed between treatment (rm ANOVA *F*_(2,105)_ = 0.477, *p =* 0.625) and treatment x time (rm ANOVA *F*_(8,105)_ = 0.351, *p =* 0.943) across the sampling period. Fig 3C displays the average number of whitefly nymphs of all stages counted on each focal tomato plant over the experiment (*n* = 8). There was no significant effect on treatment (rm ANOVA *F*_(2,105)_ = 2.72, *p =* 0.070) but there was a significant effect on treatment *x* time (rm ANOVA *F*_(8,105)_ = 2.62, *p =* 0.011). The limonene treatment had significantly less nymphs than the control on day 29 (*t =* 4.18, *df =* 105, *p <* 0.001) and neared significance at day 22 (*t =* 2.17, *df =* 105, *p =* 0.095). The limonene treatment also had significantly less whiteflies than the marigold treatment at day 29 (*t =* -2.61, *df =* 105, *p =* 0.030). Ninety five percent confidence intervals have been calculated and are available in the supporting information (S2 Dataset), but to aid visualisation they have been removed from the figure.

The effect of these treatments on settling adult whiteflies, egg and nymph numbers can be seen in Fig 3A, 3B and 3C. Across the sampling period, there was no significant effects observed on settling whitefly adults (Fig 3A) between treatment (rm ANOVA *F*_(2,105)_ = 1.71, *p =* 0.185) and treatment *x* time (rm ANOVA *F*_(8.105)_ = 0.896, *p =* 0.522) according to repeated measures ANOVA’s. Average settling whitefly numbers were lower throughout the course of the experiment on the limonene treatment (Fig 3A) whereas settling adults on the marigold treatment were actually higher than the control at day 22. This suggests that any protective effect provided by limonene dispensers is longer lasting than that provided by marigold plants. Fig 3B shows that no significant effect was observed on whitefly egg abundance between treatment (rm ANOVA *F*_(2,105)_ = 0.477, *p =* 0.625) or treatment x time (rm ANOVA *F*_(8,105)_ = 0.351, *p =* 0.943), meaning that adults were not deterred from laying eggs on the intercropped tomatoes. Whilst no significant differences were observed, average settling adults and eggs were lower on marigold and limonene intercropped plots at the second (day 7) and third (day 14) sampling point after introduction. This implies that the treatments did initially repel whitefly settling and oviposition, albeit to a non-significant extent. These results from Fig 3A and 3B would suggest that our treatments had minimal effect on whitefly behaviour and that any effects were only apparent shortly after control measures were introduced. However, there was a significant effect of treatment *x* time on whitefly nymph abundance Fig 3C across the sampling period (rm ANOVA *F*_(8,105)_ = 2.62, *p =* 0.011). Tomato plants from the limonene treatment had significantly less nymphs than the control on day 29 (*t = 4*.*18*, *df = 105*, *p < 0*.*001*) and neared significance at day 22 (*t = 2*.*17*, *df = 105*, *p = 0*.*095*). The limonene treatment also had significantly less whiteflies than the marigold treatment at day 29 (*t = -2*.*61*, *df = 105*, *p = 0*.*030*). The results from Fig 3C show that the limonene treatment had significant long-lasting effects on whitefly nymph abundance over the course of the experiment.

Taken altogether, despite the low number of significant effects between the treatments for the various whitefly life stages, these results are suggestive of a mildly suppressive effect on adult whiteflies of both marigolds and limonene (with limonene having the slightly stronger effect), and more pronounced effects on whitefly nymphs, with the limonene treatment being more effective. The fact that no significant effect was observed on settling whiteflies and egg numbers between treatments was interesting, as in previous experiments we found whiteflies to be repelled from both marigold plants and limonene dispensers. In a similar study, Du, Han [28] detected no significant mortality on different whitefly life stages, whilst the present study identified significantly fewer nymphs on tomato plants presented with limonene dispensers, which would suggest an effect on nymph mortality or egg hatch. The disparity observed in the results, of minimal effects on settling adults and eggs, but a significant effect on nymph mortality in the limonene treatment presents an interesting pattern, with several potential explanations. This may be evidence of the repellent effect being insufficiently strong to repel whiteflies completely, as they are still capable of laying as many eggs on the treated tomatoes. Toxicity of limonene against eggs or nymphs may be another explanation: limonene has been shown to be toxic to the whitefly species *Aleurodicus dispersus*, and *A*. *antidesmae*, and the inability of eggs and nymphs to move away from this chemical could make them more susceptible to any toxic effects [30]. However, in the aforementioned study from Hollingsworth [30] toxicity was not life stage specific, but achieved an equally significant effect on egg numbers.

**Fig 4:**
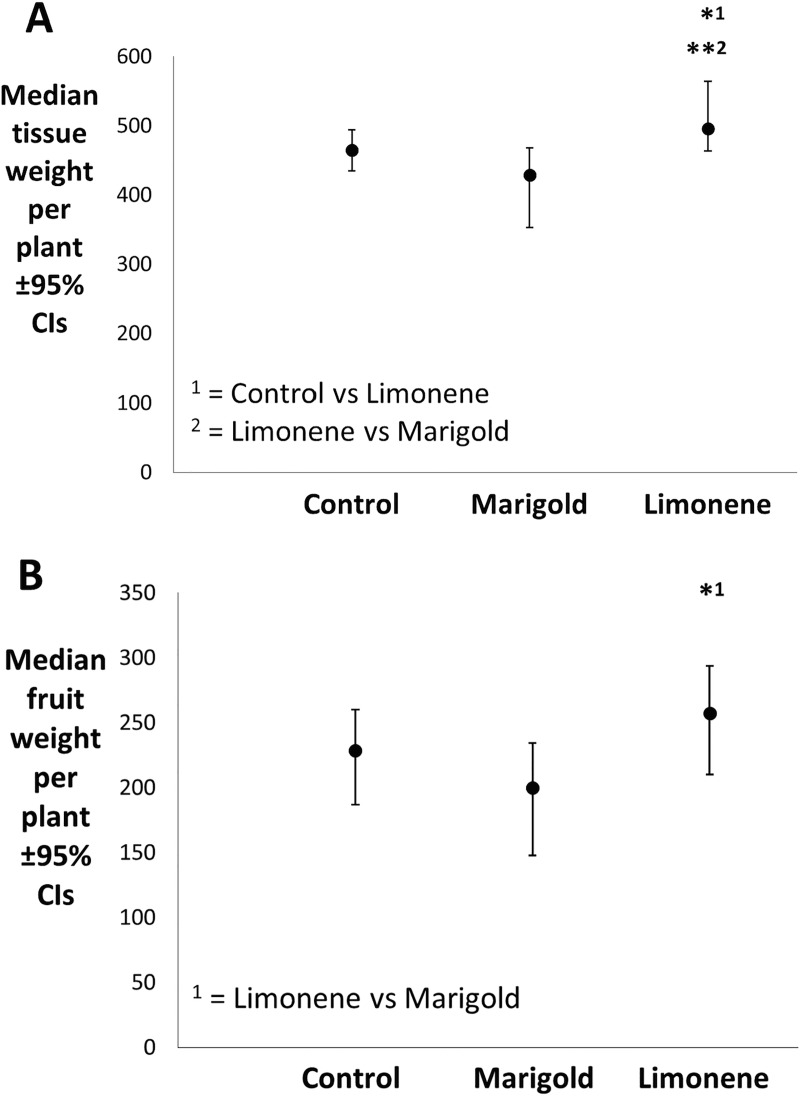
Plant development characteristics at the end of the 2017 assay into the efficacy of marigolds and limonene as an emergency treatment for the control of established whitefly populations (*n* = 8). Fig 4A displays the median aboveground tissue weight of each focal tomato plant, excluding tomatoes, at the end of the 29 day experiment (*n* = 8). Tomato plants from the marigold treatment were near-significantly lighter than tomato plants from the control (*t* = -1.86, *df* = 14, *p* = 0.085) and significantly lighter than tomato plants from the limonene treatment (*t = -3*.*3*, *df =* 14, *p* = 0.005) according to t-tests. Tomato plants from the limonene treatment were significantly heavier than those from the control according to a t-test (*t* = 2.15, *df* = 14, *p* = 0.05). Fig 4B displays the median weight of all tomatoes from each focal tomato plant at the end of the 29 day experiment (*n* = 8). The total tomato weight from plants in the marigold treatment was near-significantly lower than plants in the control (*t* = -1.76, *df* = 14, *p* = 0.100) and significantly lower than the total tomato weight of plants from the limonene treatment (*t* = -2.8, *df* = 14, *p* = 0.014). There was no significant difference in tomato weight between the control and limonene treatment.

Fig 4A and 4B show the effect of the treatments on tomato fruit production over the course of glasshouse trial 2, and the aboveground plant tissue weight (excluding tomatoes), and the total unripe tomato weight, per plant at the conclusion of the study, when plants were destructively sampled. In Fig 4A, tomato vegetative tissue in the limonene treatment was significantly heavier per plant than both the control (*p* = 0.05) and the marigold treatment (*p* = 0.005). This indicates that as a result of the limonene treatment, tomato plants were able to produce more vegetative tissue, possibly as a result of the reduction in whitefly performance on these plants. Despite this, here were no significant differences between the limonene treatment and the control in terms of total fruit weight per plant (Fig 4B). By contrast, marigold treated tomatoes experienced a near-significant reduction in fruit weight per plant compared to the control (*p* = 0.100) and a significantly lighter fruit weight than the limonene treatment (*p* = 0.014). There appears to be a difference in the effect that marigold and limonene treatments had on the plants, with limonene enhancing vegetative growth and possibly increasing fruit number, and marigold treatment potentially resulting in lighter plants with less fruit weight. An explanation for this may be found in the density of the plants in the treatments: based on observations of the plant growth in the different treatments, the introduction of marigolds (with a very bushy growth habit) may have restricted access to sunlight of the lower parts of tomato plants in that treatment, possibly inhibiting growth. The limonene treatment, by contrast, had a very low planting density, with the introduction of the limonene dispensers not restricting access to sunlight. Whilst limonene may be providing benefits to the tomato plants of reduced whitefly pest load, resulting in greater plant growth, it is necessary to see whether this advantage over controls persists in future studies that control for planting densities.

From these experiments it would appear that marigolds have a less pronounced influence on whitefly populations when introduced as an emergency control measure to reduce the impact of a heavy whitefly infestation. By contrast, application of limonene dispensers achieved a greater control over whitefly populations, giving reductions in average adult settling throughout the experiment and achieving significant reductions in nymph survival, despite similar numbers of eggs being laid on limonene-treated plants as the control. This translated to significant impacts on plant size (above ground tissue weight) and marginal impacts on fruit yield (Fig 4), showing limonene dispensers to have some degree of efficacy when deployed in this “emergency” scenario. This volatile based system involving limonene could be developed into a highly effective control agent of whitefly and since the efficacy of repellent volatiles is thought to be dose dependant [14], increasing limonene release could have a more pronounced disruptive effect. Future experiments should look to find optimum delivery levels of limonene, whilst ensuring phytotoxicity and other non-target effects do not occur.

## Conclusions and prospects

The efficacy of a method popular amongst domestic gardeners, of intercropping tomato with French marigolds for pest control, appears to have been supported in the present study. Significant control of whitefly was achieved when marigolds were intercropped amongst tomatoes from the beginning of the growth period. However introducing marigolds as a replacement for chemical control methods after significant whitefly infestation produced minimal effects. In the “emergency” situation, the limonene dispensers were more effective at reducing whitefly performance than whole plants, and this method warrants further experiments for optimisation of its deployment. Future experiments should look to ascertain how repellent volatiles effect whitefly behaviour in a no-choice situation without control plots present, which could have acted as refuge areas for the whitefly. Despite this, we predict that repellent volatiles would still reduce whitefly performance by invoking restlessness and/or abnormal behaviour, as has been shown previously [23]. Effects on natural enemies and other prominent pest species should be explored before implementing our methods in a commercial glasshouse setting. Nevertheless, the results obtained from this work are extremely promising for the application of repellent volatile chemistry for the protection of glasshouse grown tomatoes.

Increasing plant diversity further in the ‘push-pull’ assay in the first glasshouse trial did not result in enhanced pest control. Thrips, another important pest of glasshouse grown tomato, showed no level of preference to any of the treatments employed in both the “push” and “push-pull” experiments in this first glasshouse trial. In one respect this is positive and strongly suggests a future direction for this research, as it appears that other non-host plants can be added to the mixture alongside marigolds and still produce a negative effect on whiteflies on tomato, whilst having a neutral effect on other insect pests. It is envisaged that a mixture of plant species may even be developed that can be intercropped with tomato and will repel a number of the major invertebrate pests of tomato. This will be a challenge and will become more difficult as the number of pests considered increases, as each plant species must be a non-host of the focal pest, but also of the other pests the mix aims to repel (introducing a plant species that repels one pest but is suitable to another risks reducing the effectiveness of the technique). For example, both marigold [31] and tomato [32] are susceptible to the two-spotted spider mite (*Tetranychus urticae* Koch) and therefore as part of this mix a plant would need to be introduced to alleviate negative effects of this pest. We believe that through careful planning and constructing models of known plant-insect interactions it would be possible to create a mix of plants which mitigate arthropod pest damage to agricultural crops. Such a mix, if comprising edible or ornamental species, could be very attractive to growers and provide numerous societal benefits such as reducing pesticide use, diversifying horticultural production [28], increasing the diversity of invertebrate fauna within agroecosystems [13, 20], and increasing the diversity of produce on market shelves in a world increasingly dominated by fewer food types.

## Materials and methods

### Laboratory experiments

#### Whitefly

Whiteflies, *T*. *vaporariorum* (Westwood), originated from a lab culture at Rothamsted Research which was first collected in 1960 in Kent on French bean and had subsequently been maintained in a large laboratory population. The insects for both glasshouse and laboratory experiments were taken from a mixed age colony from a culture at Newcastle University (UK) where they were maintained on pre flowering aubergine (*Solanum melongena* “Moneymaker”- Marshalls Seeds Cat. 1020–2017) at 20°C, 16:8 light/dark.

#### Plants

For all laboratory experiments (Air-entrainment, free-choice and root exudates assays), tomato seeds (*S*. *lycopersicum* Mill., ‘Elegance’ Cat. E/12/11, Batch 0113479253) were obtained from Monsanto and used for all experiments. In the laboratory assays, plants were grown from seed in J. Arthur Bowers John Innes no 2 in 9-cm-diameter and 8.7-cm-deep pots. All plants were grown at a distance of approximately 60 cm from a 400-W Son-T bulb housed in a Harrier HR400SH 400-W lamp under a 16 h light/8 h dark cycle and a temperature regime of 25°C in the light and 20°C during the dark period. Tomato and marigold seedlings were grown in separate propagation units to prevent the transfer of volatile organic compounds between the two species. For air-entrainment, whitefly free-choice and root exudates assays, both tomato and marigold plants were at stage 14 on the Biologische Bundesanstalt, Bundessortenamt und Chemische Industrie (BBCH) scale [33].

#### Air entrainment volatile analysis

Headspace volatiles of marigold plants were analysed by dynamic air entrainment (Pye volatile collection kit, Kings Walden, Herts, UK) in order to deduce compounds potentially repellent to *T*. *vaporariorum*. All equipment was washed with Teepol detergent (Sigma-Aldrich) and rinsed with acetone and distilled water twice before baking at 180°C for 2 hours. Porapak Q (60/80 mesh, 0.05 g) tubes were eluted with diethyl ether (Fischer Scientific, 12347103) and heated at 140°C for 2 hr under a steam of constant nitrogen to remove contaminants, this process was repeated twice for each tube. Either a single flower or leaflet of 2–3 week old tomato seedlings was partially enclosed in a glass bell cylinder, fresh weight (g) of the entrained section was recorded after use. The bottom of the cylinder was closed without pressure around the plant stem by using two semicircular aluminum plates with a hole in the centre to accommodate the stem. Charcoal filtered air was pumped in at 1L min^−1^ and drawn out at 800 ml min^−1^ through the porapak Q adsorbent tube in a 5-mm diameter glass tube. The difference in flow rates created a slight positive pressure to ensure that unfiltered air did not enter the system, thus removing the need for an airtight seal around the stem. Plants were entrained for 24 hours from midday onwards. The porapak Q filter tube was eluted with 0.75 ml of diethyl ether (Fisher Scientific, 12347103), providing a 500μl solution that contained the isolated volatile compounds. All samples were tightly sealed in GC vials and stored at -20°C until needed.

#### Gas chromatography (GC)

GC-FID analyses were carried out using an Agilent 5890 GC. The injection port (280°C) was in the splitless mode and the flame ionization detector was heated to 300°C. The sample (1ul) in diethyl ether was injected by an HP7673 auto sampler and the split opened after 1 minute. Separation was performed on a fused silica capillary column (30m x 0.25mm i.d) coated with 0.25um dimethyl poly-siloxane (HP-5 phase). The GC was temperature programmed from 50°C-310°C at 5°C min and held at final temperature for 20 minutes with Hydrogen as the carrier gas (flow 1ml/min, pressure of 50kPa, split at 30 mls/min).

#### Gas chromatography-mass spectrometry (GC-MS)

GC-MS analysis of the biological extracts was performed on a Agilent 7890A GC split/split less injector (280°C) linked to a Agilent 5975C MSD (electron voltage 70eV, source temperature 230°C, quad temperature 150°C multiplier voltage 1200V, interface temperature 310°C). The acquisition was controlled by a HP Compaq computer using Chemstation software, initially in full scan mode (50–600 amu/sec) or in selected ion mode (30ions 0.7cps 35ms dwell) for greater sensitivity. The sample (1ul) in diethyl ether was injected by an Agilent7683B auto sampler and the split opened after 1 minute. Separation was performed on an Agilent fused silica capillary column (30m x 0.25mm i.d) coated with 0.25um dimethyl polysiloxane (HP-5) phase. The GC was temperature programmed from 50–310°C at 5°C min and held at final temperature for 10 minutes with Helium as the carrier gas (flow rate of 1ml/min, initial pressure of 50kPa, split at 30 mls/min). Peaks were identified and labelled after comparison of their mass spectra with those of the NIST05 library if > 90% fit or from their elution order from biochemical literature.

#### Limonene dispensers

The use of limonene as a treatment to control whitefly infestation required the development of a slow-release dispenser that was comparable in its rate of emission to the quantity of limonene released by a marigold plant. To achieve this, the amount of limonene released over 24h from a single marigold plant of weight 26.37g (a typical size of a marigold grown in the growth rooms at Newcastle University) was quantified as 363.26 μg (using air entrainment/GC-FID). For the lab based whitefly preference assays (Fig 2), a single 30ml medicine vial (obtained from https://bit.ly/2q9Dzwh) with a 2mm hole drilled in the screw top lid was used. This was limonene dispenser “type 1”. 3ml of pure limonene (Sigma-Aldrich, 183164) was placed inside this vial and was found to release 652.86 μg over 24 hours after headspace analysis followed by GC-MS conducted in the same way as described for the marigold seedlings. For the “heavy infestation” experiment (Fig 3), the comparable marigold plants in the glasshouse at Stockbridge Technology Centre were much larger (151.6g on average, equating to 2. 08mg of limonene per plant) than those found in the laboratory at Newcastle University and therefore limonene output needed to be increased accordingly. These same medicine vials with 3ml limonene were used, only the lid was replaced with a rectangular piece of muslin cloth secured with two elastic bands, this was limonene dispenser “type 2”. Two limonene dispensers were found to release 2.96mg of limonene over 24 hours. Limonene in the bottles was replaced every two weeks, which was shown to give a constant level of limonene output. Exactly matching emission rates to that of respective marigold plants proved difficult with the slow-release dispenser system in place here. Nevertheless, comparative limonene emission rates were achieved and future studies could invest more time developing volatile slow-release systems which exactly match volatile emissions from other repellent non-host companion plants.

#### Limonene and root exudates free-choice assays

To confirm the repellent effect of marigold and limonene to adult *T*. *vaporariorum*, a series of laboratory bioassays were conducted. For each experiment 8 “Elegance” tomato seedlings were placed inside a 90x60x60cm mesh cage (Watkins and Doncaster, product code: E6098). These 8 plants were separated into two groups of four and placed at opposite ends of the cage, 50cm apart. A schematic overview of this setup can be seen in the S7 Fig. Two-hundred whiteflies of mixed age and sex were released into the centre of the cage and total eggs and settling adults on every plant was recorded after exactly 24 hours. For the repellence assay, two marigold seedlings were placed in the two corners of the cage behind one of the two groups of tomato seedlings. Whitefly preference was assessed based on their location in the cage, i.e. at the side accompanied by marigold seedlings or not. The same procedure was conducted for “type 1” limonene dispensers and each experiment was repeated a total of 6 times.

To assess the effect of root exudate sharing between tomato and marigold, similar whitefly free-choice assays were conducted where both species were placed in communal drip trays (52x42cm) prior to assessment of whitefly settling and oviposition preference. Four tomato seedlings were placed in small plastic drip tray filled with water to make the pots roughly 2cm submerged, topping up when necessary. Two flowering marigold seedlings were introduced to these drip trays for one or seven days, and for control replicates 2 tomato seedlings were added. Whilst plants in the glasshouse study shared root exudates for longer, difficulties in replicating this with the laboratory growth space available limited us to these time frames. Despite this, it was assumed that any morphological and/or physiological changes resulting from root exudate sharing would be apparent after the time frames implemented here. These plants remained in the growth rooms under the Harrier HR400SH 400W lamp described previously. Whitefly preference was then assessed against four other tomato seedlings (taken straight from the growth rooms described previously) and set up in the same 90x60x60 cm cage as described previously. For both experiments the Pearsons chi-squared test was used to compare settling distribution and expected values for the marigold and limonene assays were assumed as equal (50/50). For the root exudates assays, settling distribution was compared to the average across the 6 control replicates described above.

#### Glasshouse experiment 1: “Push”, “Push-Pull” and “High Diversity” control strategies applied at the beginning of the infestation period

In order to establish a baseline measure of whitefly preference for different plant species, a laboratory assay was conducted using plant leaf disks in a no choice assay that quantified *T*. *vaporariorum* preference for each species individually. On the basis of the leaf disk results nasturtium, Chinese cabbage, basil, and marigold were designated as ‘push’ plants and pumpkin, melon, courgette, and sunflower as ‘pull’ plants in the glasshouse trial that followed. S6 Fig displays the full results of the leaf disc assays with comparisons to similar experiments on *T*. *vaporariorum* preference from published literature.

Experiments were conducted in the mid-late growing season (started 10^th^ August) 2016 in a 448m^3^ glasshouse in the grounds of Stockbridge Technology Centre Ltd., Yorkshire, England (Grid Ref. SE 55977 36605). Experiments were supplemented with a population of *T*. *vaporariorum* consisting of 3 heavily infested aubergine (*Solanum melongena* “Moneymaker”) plants from the culture described above, distributed in the centre of the greenhouse. The glasshouse represented a closed environment which limited the ability of natural pests to infest our experimental crop. However, the glasshouse was not screened and we expect that some whiteflies from the surrounding environment did infest our experimental crop, but that the vast majority originated from the introduced cultures described. We did observe low numbers of thrips and spider mites (which entered the glasshouse naturally) in both glasshouse trials. Plants were grown from seed, one per compartment, in standard seed germination trays in glasshouses at Stockbridge Technology Centre (UK) for 5 weeks. At the point of replanting for both “push” and “push-pull” experiments, plants were at stage 13 on the BBCH scale and had the following number of fully expanded leaves: tomatoes 3–6 leaves, marigold 4–6 leaves, basil 3–4 leaves, nasturtium 4–5 leaves, cabbage 4–6 leaves, sunflower 3–5 leaves and courgette, pumpkin, and melon 2–4 leaves. Plants were replanted into 5 litre pots and placed into 110 x 55 x 4cm drip trays, 8 pots to a tray. Plants were replanted in Clover Multipurpose Compost (Dungannon, Co. Tyrone,N, Ireland BT71 4QR).

Two experiments were undertaken to investigate whether intercropping from the start of the growth period could assert control over whitefly populations, S8 Fig contains a schematic overview of these experiments. The first experiment sought to answer whether intercropping with marigolds could reduce whitefly numbers on tomato by ‘pushing’ whiteflies from tomato, and if so, whether this effect could be enhanced by the use of additional non-host, ‘push’ plants. The second experiment sought to identify whether the ‘push’ effect could be combined with the ‘pull’ effect of attractive host plants placed around the edge of the treatment, to attract whiteflies from tomatoes, and whether this effect could be further intensified by using a higher diversity of both ‘push’ and ‘pull’ plant species.

Single, fully-expanded leaves were selected by randomising both compound leaf selection and then individual leaflet from 8 tomato plants per replicate. These were then examined *in situ* for *T*. *vaporariorum* adults and adults of other insect pests. The sampling regime can be viewed in Fig 1 and covered a period of 48 days. These leaves were removed and placed in sealed plastic bags, then stored overnight at 4°C and examined the next day for whitefly (and other pest) larvae and eggs. The abundance of all pest insects present was recorded to test the effect of intercropping on other pest species as well as the target pest *T*. *vaporariorum*. *Thrips tabaci* were the only other pest to be consistently observed in the glasshouse, albeit at very low numbers. Other insects were encountered in very low abundances so were not included in analysis. Thrips population development in both experiments is presented as average number of pests per leaf and can be viewed in S1 Fig. Procedures were identical for the ‘push-pull’ experiment, the outer ring of pull plants was not sampled. At the end of the experimental period, each focal tomato plant was destructively sampled and total fruit and total aboveground tissue weight measured (S2 Fig).

Data for insect abundance was non-normally distributed and was therefore (log + 1) transformed to meet normality and homogeneity of variance assumptions for statistical analysis. Whitefly and thrips abundance (adult, larvae and egg numbers were added together to give a single value for each insect) were analysed with repeated measures ANOVA’s using the “lmerTest” package in R. Time (sampling date) was used as the repeated measure with treatment and the treatment x time interaction as fixed factors. The Bonferroni post-hoc correction was used to analyse differences between treatments at individual sampling points, this was done by multiplying *p* values by the total number of comparisons made. Therefore all *p* values reported are significant at *α* = 0.05. Tomato plant and fruit weights were also non-normally distributed but were analysed with Mann Whitney U tests. The non-parametric effect size measure, Cliff’s delta (*d*) was determined using the ‘effsize’ package for R in order to allow a standardised comparison of effects that takes into account different start times of experiments [34]. The Cliff’s delta *(d)* measure was ascertained from the un-transformed non-parametric data.

#### Glasshouse Experiment 2: “Emergency” Intercropping to protect tomatoes from an established heavy whitefly infestation

These experiments were run in the same greenhouse at Stockbridge Technology centre from 13^th^ July 2017 – 14^th^ August 2017. The aim of the experiment was to build up a high density whitefly population on a tomato crop, then introduce either more tomatoes (control), French marigolds (*T*. *patula*; marigold treatment), or limonene dispensers (limonene treatment), and observe the level of whitefly control achieved. Marigolds and tomatoes for this experiment were planted as seeds in standard seed germination trays in a glasshouse at Stockbridge Technology Centre on 25^th^ April 2017. On 6^th^ June 2017, when the plants had reached stage 13 on the BBCH scale, plants were re-potted in the experimental glasshouse into 5L pots containing 5L of Clover multipurpose compost (details above). These plants were then divided into two groups: 192 tomato plants (hereafter referred to as the focal tomato plants) were placed in the experimental glasshouse in 110 x 55 x 4cm drip trays as described above, and were subdivided into three treatment blocks, replicated eight times. A schematic overview of both experiments in the glasshouse can be viewed in S8 Fig. These plants had four heavily infested aubergine plants from the laboratory at Newcastle University placed amongst them to supplement naturally occurring whitefly pest populations (S8 Fig); the aim was to achieve a heavy whitefly infestation across the whole glasshouse. The second group of plants (comprising 64 tomato plants and 64 marigold plants) were placed in an adjacent greenhouse, of the same dimensions as the experimental greenhouse, and covered in porous white gauze. The aim was to keep these plants uninfested so as to not affect the whitefly population once they were introduced.

Both groups of plants were grown in their respective greenhouses for 13 days until 26^th^ July 2017, at which point a high density whitefly population had been achieved (this density may be seen in the first sampling point of Fig 3A). On this date, the uninfested plants from the second greenhouse were introduced into the infested greenhouse, and arranged amongst their respective treatments as follows. The experiment was arranged as for the ‘Push’ experiment outlined (see S8 Fig) above, with eight replicates of three treatments: a control, a marigold treatment and a limonene treatment (which was used in place of the HD treatment, see S8 Fig). The control, as before, comprised eight, whitefly-infested focal tomato plants, with eight uninfested tomato plants randomly distributed amongst them. The marigold treatment comprised eight focal tomato plants with eight marigold plants randomly distributed amongst them (identical to the LD ‘push- treatment, see S8 Fig). The limonene treatment included eight tomato plants with 16 “type 2” limonene dispensers (described above) bottles randomly distributed amongst them, with limonene bottles placed two per 5L pot filled with 5L of soil.

After the introduction of the treatment plants, the experiment was continued for a further 29 days, with sampling being undertaken in the same way as for the ‘push-pull’ assay. The number of adult insects, including whitefly adults, which had settled on a single leaf from each of the focal tomato plants were counted each week, as well as the number of unripe tomatoes as they emerged. As before, adult insects were assessed on the day, and eggs and nymphs counted the next day under low power microscopy. Plants were sampled weekly over 29 days, after which the experiment was ended due to declining plant health in the greenhouse, possibly due to the heavy whitefly infestation. At the culmination of the experiment, after the insect assessment, each focal plant was destructively sampled and total fruit and total aboveground tissue weight measured. Very few tomatoes were ripe on each treatment, so tomato weights represent the weight of the green tomatoes on the plants at the time of harvesting. Abundance of all whitefly life stages was (log +1) transformed and analysed with repeated measures ANOVA’s with Bonferroni post-hoc correction in the instance of a significant interaction (as above in glasshouse trial 1). The final plant and fruit weight were compared using t-tests on untransformed data.

## Supporting information

S1 Fig Thrips population development from glasshouse trial 1. Population development of thrips (*T*. *tabaci*) on tomato in the glasshouse, with all thrips life stages (adult and larvae) contributing to the average number of thrips/leaf. Data was (log +1) transformed and repeated measures ANOVA’s were used to assess the effect of treatment and treatment *x* time on thrips abundance across the experimental period. No significant interactions were observed between treatments (rm ANOVA *F*_(2,126)_ = 0.95, *p =* 0.388) or treatment *x* time (rm ANOVA *F*_(10,126)_ = 0.570, *p =* 0.835) for the “push” experiment (A). For the “push-pull” experiment (B), no significant effects were observed between treatment (rm ANOVA *F*_(2,105)_ = 0.333, *p =* 0.717) and treatment *x* time (rm ANOVA *F*_(8,105)_ = 0.420, *p =* 0.906). Ninety five percent confidence intervals have been calculated and are available in the supplementary information (S1 Dataset), but to aid visualisation they have been removed from the figure. S1A Fig shows the “push” experiment in which repellent plants such as marigold (LD) and marigold and other non-hosts (HD) are distributed amongst tomato plants. S1B Fig shows the “push-pull” experiment which is the same as the “push” experiment but additionally a single (LD) and several (HD) host plant species are placed around the perimeter of the mixture of repellent hosts and tomato.
(PDF)

S2 Fig Plant development characteristics from “push” and “push-pull” experiments in glasshouse trial 1. Plant development characteristics at the end of the 2016 “push” and “push-pull” glasshouse trials (*n* = 8). S2A Fig shows the median above ground plant weight for each of the three treatments (control, low diversity and high diversity) from the “push” experiment with 95% confidence intervals annotated as error bars. S2B Fig shows the median weight of tomatoes per plant per treatment also from the “push” experiment, 95% confidence intervals are annotated as error bars. S2C Fig shows the median above ground plant weight for each treatment in the “push-pull” experiment with 95% confidence intervals annotated as error bars. There is no tomato yield data for the “push-pull” experiment as tomatoes had not yet formed due to this experiment starting later than the “push” experiment and being subsequently cut-short by the onset of late season blight. The Mann Whitney *U* test was used to compare differences between the treatments as the data was found to be non-normally distributed. For all three graphs, there was no significant differences observed between any of the treatments.
(PDF)

S3 Fig Whitefly repellence assays: Sharing of root exudates. The percentage of settling whiteflies (*n* = 200) when given the choice between four “Elegance” tomato seedlings or four “Elegance” seedlings previously cultivated in communal drip trays with flowering *T*. *patula* seedlings for either 24 hours or 7 days (S3A and S3B Fig). The tomato plants which were grown in this way are referred to with the acronym SRE (shared root exudates). Whitefly eggs were also recorded for each replicate (S3C and S3D Fig) and the Pearson’s chi-squared test was used to test differences in settling and oviposition behaviour compared to control distributions. Significant differences are annotated onto the graph for each treatment with the following format; *p < 0.05 significance; **p < 0.01 significance, *df* = 1 for all groups. For S3A Fig, rep 1 *X* ^2^ = 0.18, *p* = 0.671; rep 2 *X* ^2^ = 3.92, *p* = 0.048; rep 3 *X* ^2^ = 0.08, *p* = 0.776; rep 4 *X* ^2^ = 0.72, *p* = 0.396; rep 5 *X* ^2^ = 0.02, *p* = 0.887; rep 6 *X* ^2^ = 0.98, *p* = 0.332. For S3B Fig, rep 1 *X* ^2^ = 1.64, *p* = 0.199; rep 2 *X* ^2^ = 0.02, *p* = 0.887; rep 3 *X* ^2^ = 0.500, *p* = 0.479; rep 4 *X* ^2^ = 0.20, *p* = 0.887; rep 5 *X* ^2^ = 0.504, *p* = 0.478; rep 6 *X* ^2^ = 0.320, *p* = 0.572. For S3C Fig, rep 1 *X* ^2^ = 1.62, *p* = 0.202; rep 2 *X* ^2^ = 0.082, *p* = 0.775; rep 3 *X* ^2^ = 0.02, *p* = 0.886; rep 4 *X* ^2^ = 7.22, *p* = 0.007; rep 5 *X* ^2^ = 0.325, *p* = 0.569; rep 6 *X* ^2^ = 0.021, *p* = 0.886. For S3D Fig, rep 1 *X* ^2^ = 0.08, *p* = 0.777; rep 2 *X* ^2^ = 2.04, *p* = 0.153; rep 3 *X* ^2^ = 1.64, *p* = 0.203; rep 4 *X* ^2^ = 6.61, *p* = 0.010; rep 5 *X* ^2^ = 1.64, *p* = 0.199; rep 6 *X* ^2^ = 0.322, *p* = 0.570. On average over the 6 replicates, 49.89% of whiteflies settled on SRE tomato in the 24 hour treatment and 49.91% whiteflies settled on SRE tomato in the 7 day treatment.
(PDF)

S4 Fig Limonene identification and quantification. S4A Fig, GC-FID profile showing the volatile output from a single French marigold flower over 24h. For both marigold flowers and leaf tissue, limonene was the most prominent volatile and the GC trace shown was typical across all the replicates. Limonene formed 24.01% and 21.04% of the volatile output from both flowers and leaf tissue respectively. Whilst other prominent volatiles were detected in marigold headspace samples, none matched the emission rates and cost effective appeal which was offered through the use of limonene. It was therefore decided that whitefly repellence to this individual chemical would be assessed. Other identified volatiles from the GC-FID trace are labelled concurrently, “^” indicates confirmation of presence with authentic standards, where available; 1 = limonene^, RT 11.91; 2 = α-pinene^, RT 8.37; 3 = isobutyric acid, RT 11.45; 4 = (*E*/*Z*)-β-ocimene^, RT 12.66; 5 = Siloxane contaminant (originating from the GC column), RT 16.05; 6 = terpinolene^, RT 17.03; 7 = Butylated hydroxytoluene (stabilising agent from the diethyl ether), RT 25.95. S4B Fig displays average limonene release from marigold flowers and leaf tissue was quantified and displayed as μg of limonene per gram of fresh weight over 24 hours. Error bars display upper and lower bound 95% confidence intervals, each tissue group was replicated 5 times.
(PDF)

S5 Fig Whitefly repellence assay: Four-way olfactometer. Whitefly response to marigold plant tissue and limonene was tested using a 4-way olfactometer with four different treatments; one marigold flower, marigold leaves that were the same weight as one marigold flower (1.713g) (ML1), marigold leaves that were double the weight of one marigold flower (3.426g) (ML2) and a “type 1” limonene dispenser. The two different amounts of leaf tissue were chosen considering the amount of limonene released from flowers was approximately double that of leaf tissue per gram of fresh weight (S4B Fig). For each treatment, 100 whiteflies of mixed sex were introduced to the olfactometer and average (n = 4) percentage of whiteflies which selected the wing of the olfactometer containing the marigold plant tissue or a limonene dispenser is displayed. The Pearson’s chi-squared test was used to test if distribution of whiteflies differed significantly from the average settling distribution across 4 control replicates where only tomato was present in all four wings of the olfactometer. Significant differences are annotated onto the graph with “*”. For each of the treatments; marigold flower = *X* ^2^ = 11.21, *p* = 0.001; ML1 treatment = *X* ^2^ = 0.653, *p* = 0.419; ML2 = *X* ^2^ = 8.87, *p* = 0.001; limonene dispenser = *X* ^2^ = 6.33, *p* = 0.050, *df* = 3 for all. Average settling percentages across the four experimental wings of the olfactometer in the control was 24.70%. For each of the treatments, average settling in wings containing individual treatment materials were as follows; marigold flower = 10.5%, ML1 = 21.5%. ML2 = 12.1%, limonene = 14.1%. Methods for this experiment can be found in S1 materials and methods.
(PDF)

S6 Fig Whitefly host preference leaf disc assays. The non-choice plant tissue preference assay may be seen in S6A Fig, with 8 disks of tomato in each dish. Fifty whiteflies were added and the average number of whiteflies settled on plant tissue after 21h was calculated. Average whiteflies settled (*n* = 8) on each plant species can be seen in S6B Fig, with 95% confidence intervals for the mean plotted, and the species ordered in order of preference. This quantification of preference agreed with previous broad surveys of *T*. *vaporariorum* plant range (S6C Fig) from CABI [35], Roditakis [36] and Mound and Halsey [37], respectively, with non-hosts being less preferred and hosts more preferred. ‘+’ indicates ‘host’, ‘-’ indicates ‘non-host’ and ‘0’ indicates that this plant was not considered. Methods for this experiment can be found in S1 materials and methods.
(PDF)

S7 Fig Schematic overview of whitefly free choice assays. This diagram shows a plan view of the experimental design employed for the whitefly free-choice assays.
(PDF)

S8 Fig Schematic overview of glasshouse experiments. Layout of experiments to test the efficacy of ‘push’ and ‘push-pull’ strategies against the glasshouse whitefly on tomato, and the efficacy of intercropping at reducing large whitefly population sizes. A randomised block design was used, with each of the 8 replicates containing all 3 treatments for each study in a random order. The ‘push’ experiment involved intercropping tomato with non-hosts. The ‘push-pull’ experiment was similar but additionally had attractive host plants around the perimeter. Whitefly plant preference for the various hosts was determined in laboratory leaf disk experiments (S6 Fig) and confirmed by literature surveys. The location of heavily infested aubergine plants used to supplement natural whitefly populations are shown with triangles containing the letter ‘W’. The experiment to test the introduction of plants during an advanced whitefly infestation was nearly identical in layout to the ‘Push’ experiment, but with limonene dispensers placed in compost replacing the non-tomato plant species in the HD treatment used the ‘push’ assay. Acronyms for the treatments used are as follows; C—control, LD—low diversity, HD—high diversity. For the individual plants within each treatment; T—tomato, M—marigold, N—nasturtium, B—Basil, C–Chinese cabbage, Me–Melon, Co–courgette, S–sunflower.
(PDF)

S1 Dataset Raw data from glasshouse experiment 1. (XLSX)

S2 Dataset Raw data from glasshouse experiment 2. (XLSX)

S1 Materials and Methods Materials and methods for supporting information files S5 Fig and S6 Fig. (DOCX)
