# Supplementary figures and images for: Companion planting with French marigolds protects tomato plants from glasshouse whiteflies through the emission of airborne limonene
Source: PLoS One. 2019 Mar 1;14(3):e0213071. doi: 10.1371/journal.pone.0213071 (PMC6396911; doi:10.1371/journal.pone.0213071)

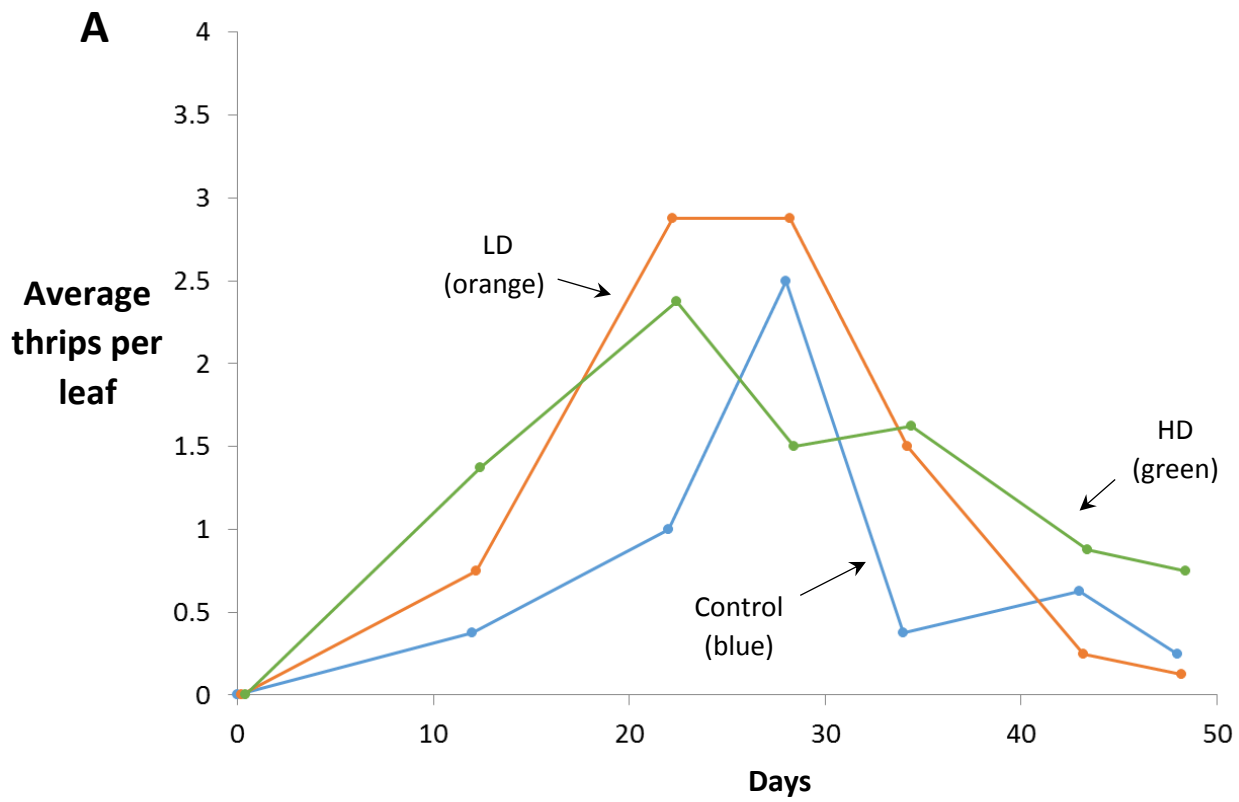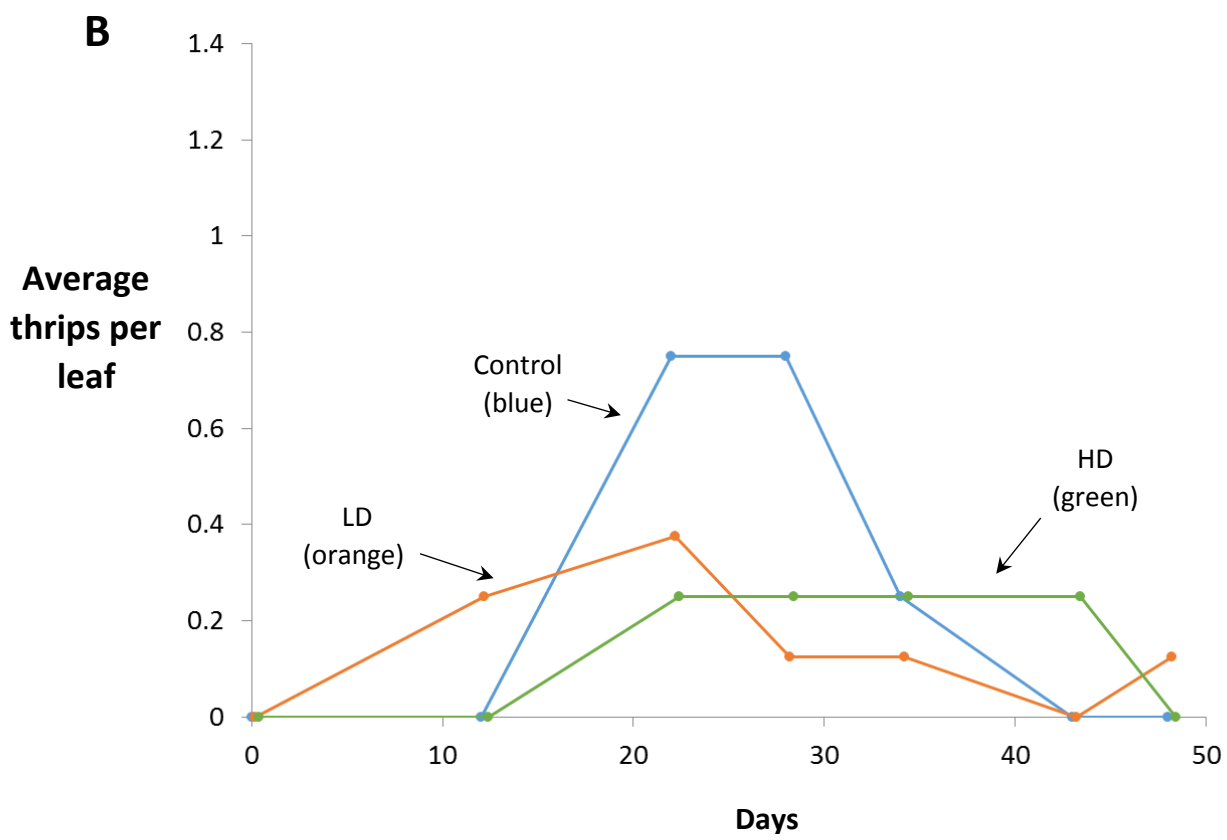

Supplement: S1 Fig — Population development of thrips (T. tabaci) on tomato in the glasshouse, with all thrips life stages (adult and larvae) contributing to the average number of thrips/leaf. Data was (log +1) transformed and repeated measures ANOVA’s were used to assess the effect of treatment and treatment x time on thrips abundance across the experimental period. No significant interactions were observed between treatments (rm ANOVA F (2,126) = 0.95, p = 0.388) or treatment x time (rm ANOVA F (10,126) = 0.570, p = 0.835) for the “push” experiment (A). For the “push-pull” experiment (B), no significant effects were observed between treatment (rm ANOVA F (2,105) = 0.333, p = 0.717) and treatment x time (rm ANOVA F (8,105) = 0.420, p = 0.906). Ninety five percent confidence intervals have been calculated and are available in the supplementary information (S1 Dataset), but to aid visualisation they have been removed from the figure. S1A Fig shows the “push” experiment in which repellent plants such as marigold (LD) and marigold and other non-hosts (HD) are distributed amongst tomato plants. S1B Fig shows the “push-pull” experiment which is the same as the “push” experiment but additionally a single (LD) and several (HD) host plant species are placed around the perimeter of the mixture of repellent hosts and tomato. (PDF) [file pone.0213071.s001.pdf]

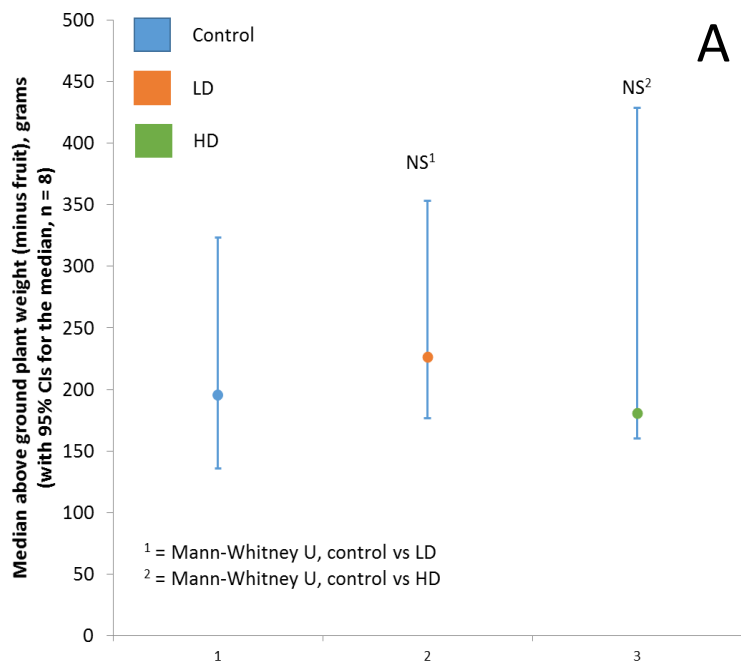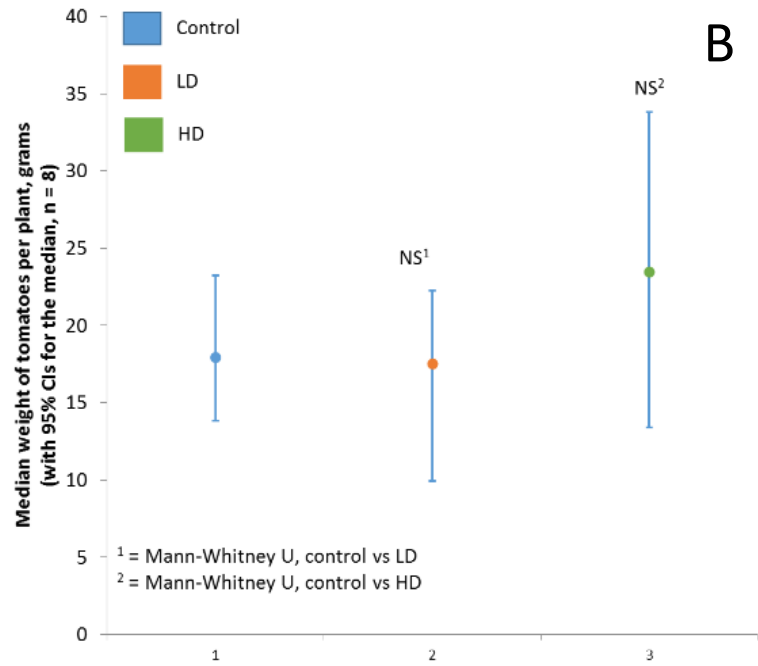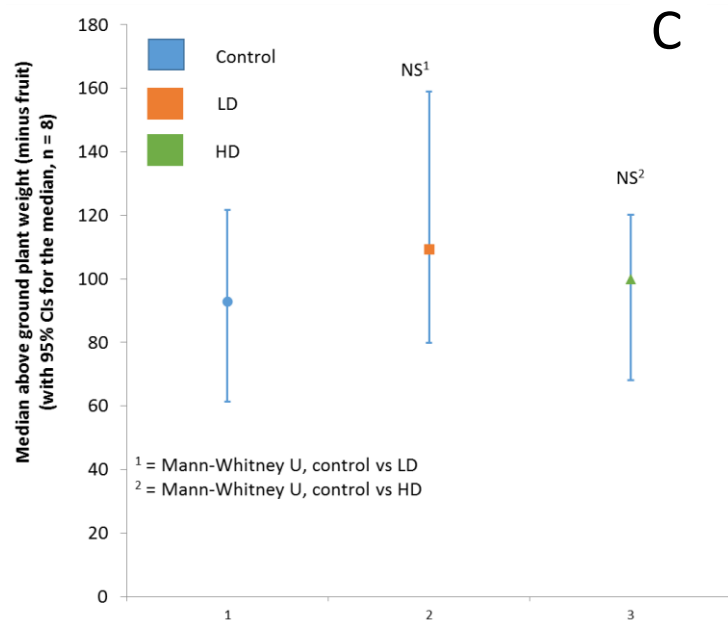

Supplement: S2 Fig — Plant development characteristics at the end of the 2016 “push” and “push-pull” glasshouse trials (n = 8). S2A Fig shows the median above ground plant weight for each of the three treatments (control, low diversity and high diversity) from the “push” experiment with 95% confidence intervals annotated as error bars. S2B Fig shows the median weight of tomatoes per plant per treatment also from the “push” experiment, 95% confidence intervals are annotated as error bars. S2C Fig shows the median above ground plant weight for each treatment in the “push-pull” experiment with 95% confidence intervals annotated as error bars. There is no tomato yield data for the “push-pull” experiment as tomatoes had not yet formed due to this experiment starting later than the “push” experiment and being subsequently cut-short by the onset of late season blight. The Mann Whitney U test was used to compare differences between the treatments as the data was found to be non-normally distributed. For all three graphs, there was no significant differences observed between any of the treatments. (PDF) [file pone.0213071.s002.pdf]

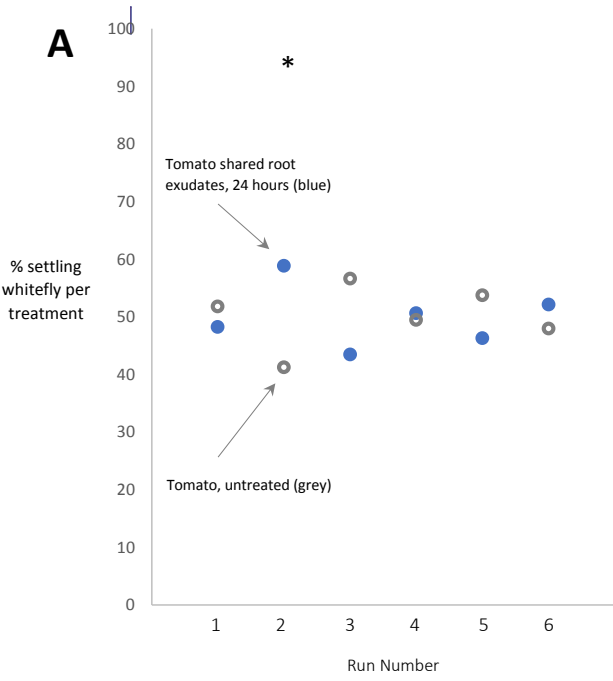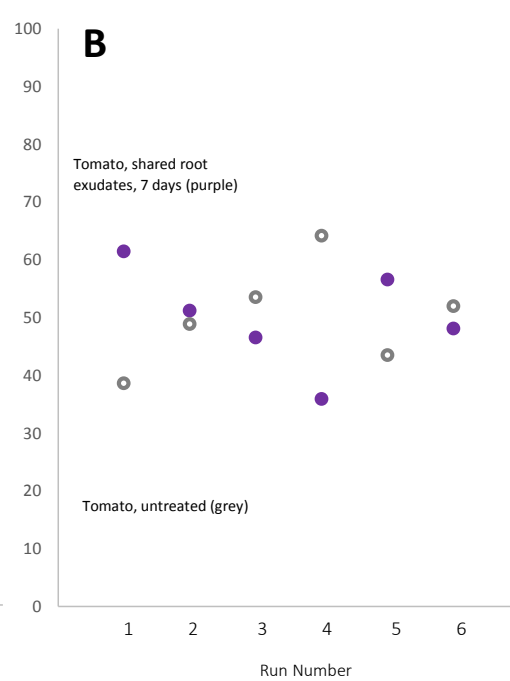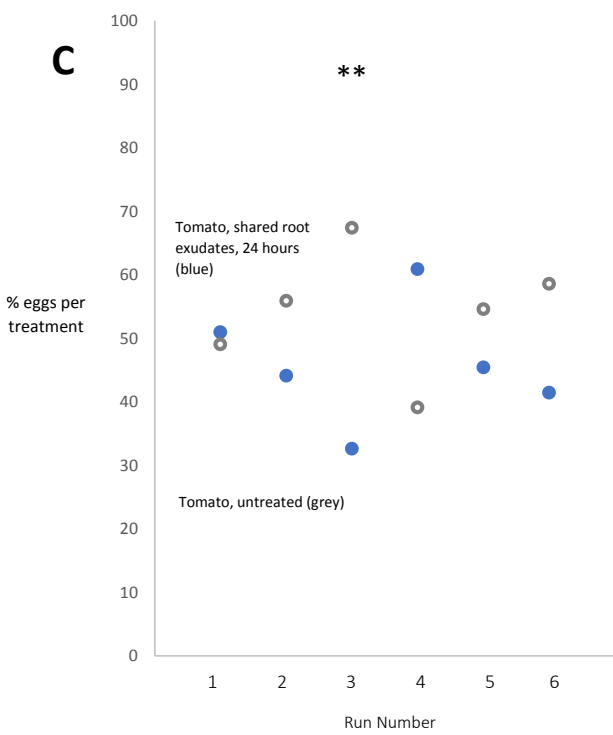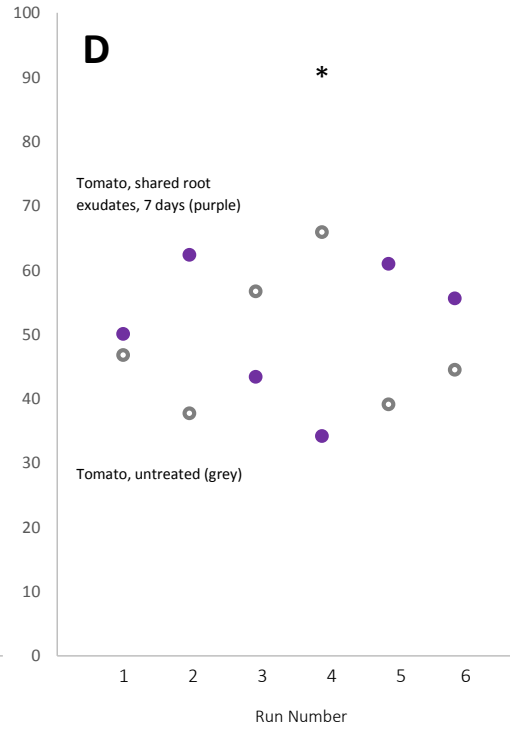

Supplement: S3 Fig — The percentage of settling whiteflies (n = 200) when given the choice between four “Elegance” tomato seedlings or four “Elegance” seedlings previously cultivated in communal drip trays with flowering T. patula seedlings for either 24 hours or 7 days (S3A and S3B Fig). The tomato plants which were grown in this way are referred to with the acronym SRE (shared root exudates). Whitefly eggs were also recorded for each replicate (S3C and S3D Fig) and the Pearson’s chi-squared test was used to test differences in settling and oviposition behaviour compared to control distributions. Significant differences are annotated onto the graph for each treatment with the following format; *p < 0.05 significance; **p < 0.01 significance, df = 1 for all groups. For S3A Fig, rep 1 X 2 = 0.18, p = 0.671; rep 2 X 2 = 3.92, p = 0.048; rep 3 X 2 = 0.08, p = 0.776; rep 4 X 2 = 0.72, p = 0.396; rep 5 X 2 = 0.02, p = 0.887; rep 6 X 2 = 0.98, p = 0.332. For S3B Fig, rep 1 X 2 = 1.64, p = 0.199; rep 2 X 2 = 0.02, p = 0.887; rep 3 X 2 = 0.500, p = 0.479; rep 4 X 2 = 0.20, p = 0.887; rep 5 X 2 = 0.504, p = 0.478; rep 6 X 2 = 0.320, p = 0.572. For S3C Fig, rep 1 X 2 = 1.62, p = 0.202; rep 2 X 2 = 0.082, p = 0.775; rep 3 X 2 = 0.02, p = 0.886; rep 4 X 2 = 7.22, p = 0.007; rep 5 X 2 = 0.325, p = 0.569; rep 6 X 2 = 0.021, p = 0.886. For S3D Fig, rep 1 X 2 = 0.08, p = 0.777; rep 2 X 2 = 2.04, p = 0.153; rep 3 X 2 = 1.64, p = 0.203; rep 4 X 2 = 6.61, p = 0.010; rep 5 X 2 = 1.64, p = 0.199; rep 6 X 2 = 0.322, p = 0.570. On average over the 6 replicates, 49.89% of whiteflies settled on SRE tomato in the 24 hour treatment and 49.91% whiteflies settled on SRE tomato in the 7 day treatment. (PDF) [file pone.0213071.s003.pdf]

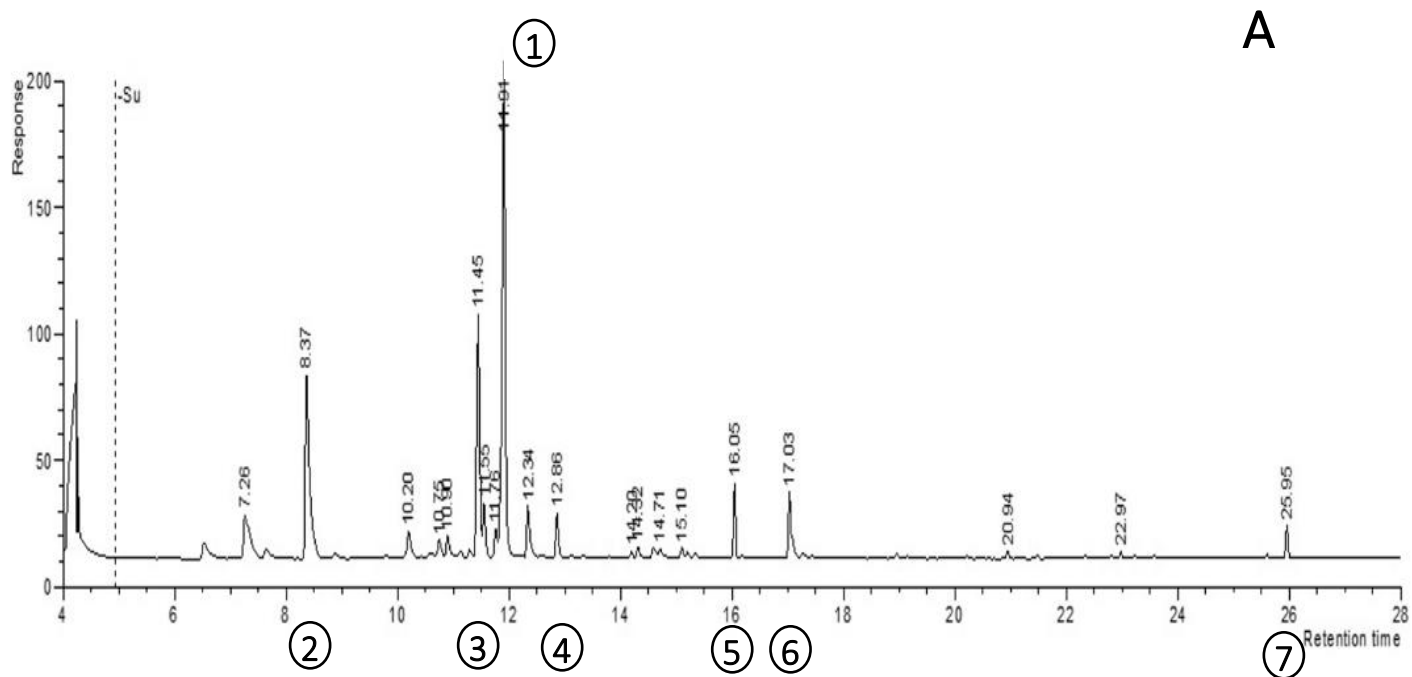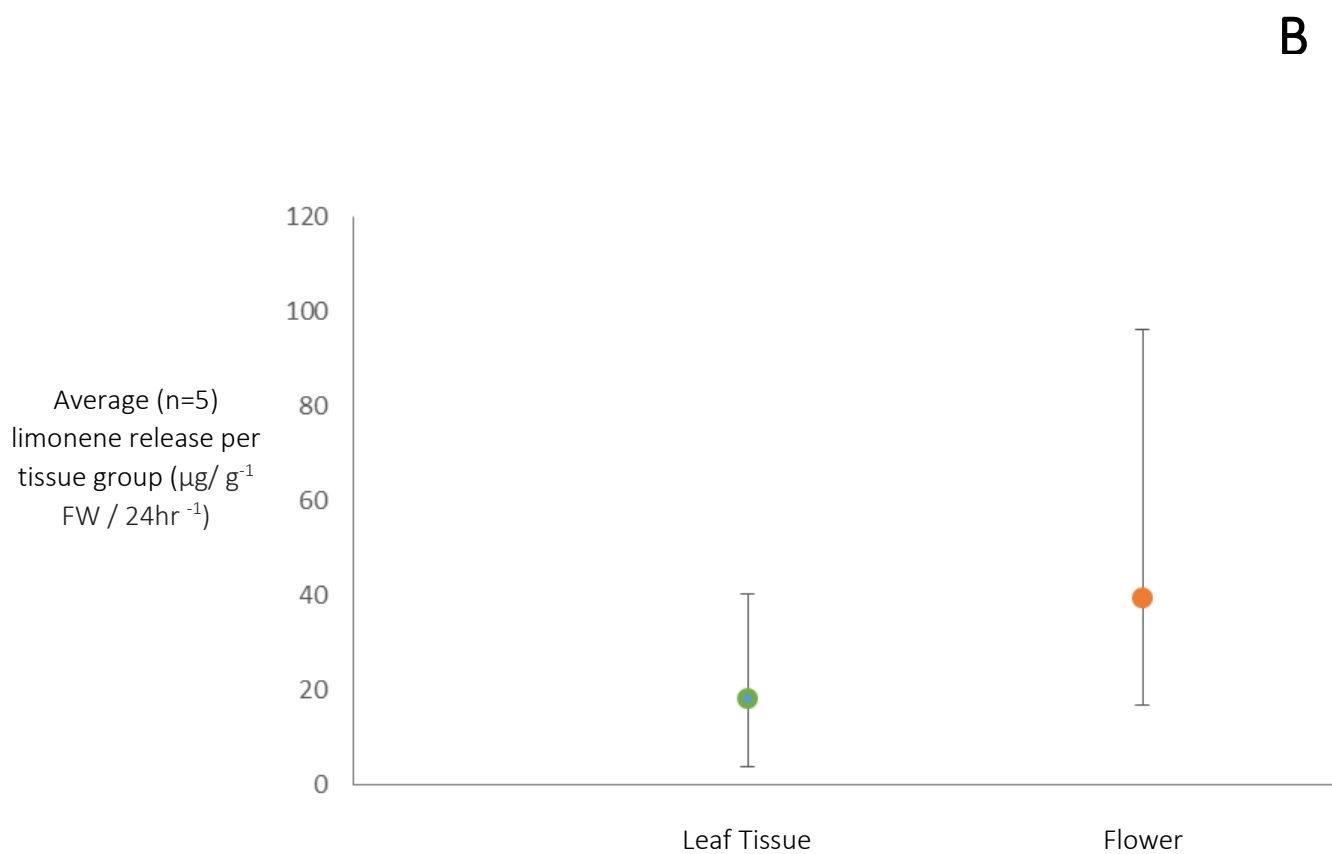

Supplement: S4 Fig — S4A Fig, GC-FID profile showing the volatile output from a single French marigold flower over 24h. For both marigold flowers and leaf tissue, limonene was the most prominent volatile and the GC trace shown was typical across all the replicates. Limonene formed 24.01% and 21.04% of the volatile output from both flowers and leaf tissue respectively. Whilst other prominent volatiles were detected in marigold headspace samples, none matched the emission rates and cost effective appeal which was offered through the use of limonene. It was therefore decided that whitefly repellence to this individual chemical would be assessed. Other identified volatiles from the GC-FID trace are labelled concurrently, “^” indicates confirmation of presence with authentic standards, where available; 1 = limonene^, RT 11.91; 2 = α-pinene^, RT 8.37; 3 = isobutyric acid, RT 11.45; 4 = (E/Z)-β-ocimene^, RT 12.66; 5 = Siloxane contaminant (originating from the GC column), RT 16.05; 6 = terpinolene^, RT 17.03; 7 = Butylated hydroxytoluene (stabilising agent from the diethyl ether), RT 25.95. S4B Fig displays average limonene release from marigold flowers and leaf tissue was quantified and displayed as μg of limonene per gram of fresh weight over 24 hours. Error bars display upper and lower bound 95% confidence intervals, each tissue group was replicated 5 times. (PDF) [file pone.0213071.s004.pdf]

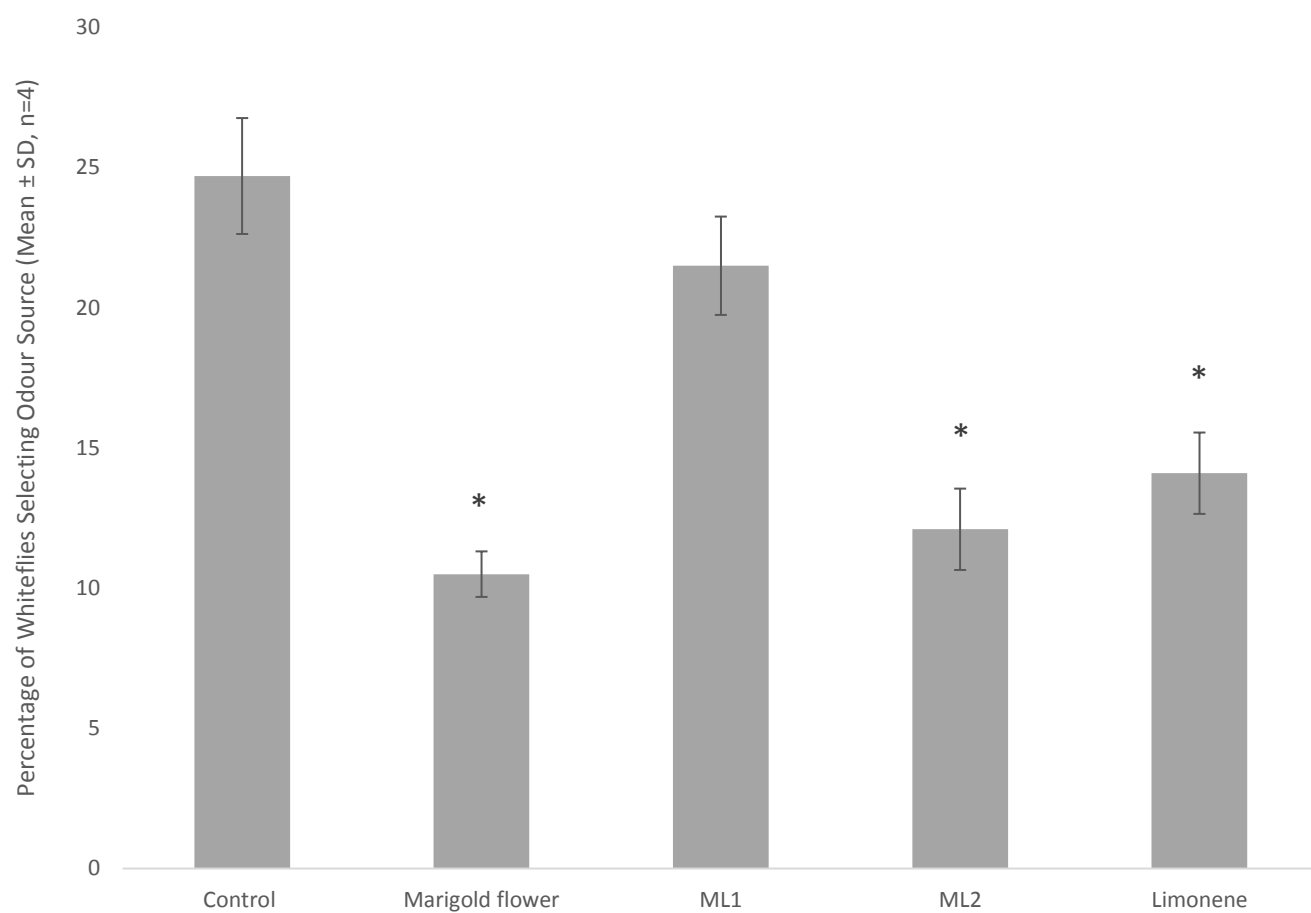

Supplement: S5 Fig — Whitefly response to marigold plant tissue and limonene was tested using a 4-way olfactometer with four different treatments; one marigold flower, marigold leaves that were the same weight as one marigold flower (1.713g) (ML1), marigold leaves that were double the weight of one marigold flower (3.426g) (ML2) and a “type 1” limonene dispenser. The two different amounts of leaf tissue were chosen considering the amount of limonene released from flowers was approximately double that of leaf tissue per gram of fresh weight (S4B Fig). For each treatment, 100 whiteflies of mixed sex were introduced to the olfactometer and average (n = 4) percentage of whiteflies which selected the wing of the olfactometer containing the marigold plant tissue or a limonene dispenser is displayed. The Pearson’s chi-squared test was used to test if distribution of whiteflies differed significantly from the average settling distribution across 4 control replicates where only tomato was present in all four wings of the olfactometer. Significant differences are annotated onto the graph with “*”. For each of the treatments; marigold flower = X 2 = 11.21, p = 0.001; ML1 treatment = X 2 = 0.653, p = 0.419; ML2 = X 2 = 8.87, p = 0.001; limonene dispenser = X 2 = 6.33, p = 0.050, df = 3 for all. Average settling percentages across the four experimental wings of the olfactometer in the control was 24.70%. For each of the treatments, average settling in wings containing individual treatment materials were as follows; marigold flower = 10.5%, ML1 = 21.5%. ML2 = 12.1%, limonene = 14.1%. Methods for this experiment can be found in S1 materials and methods. (PDF) [file pone.0213071.s005.pdf]

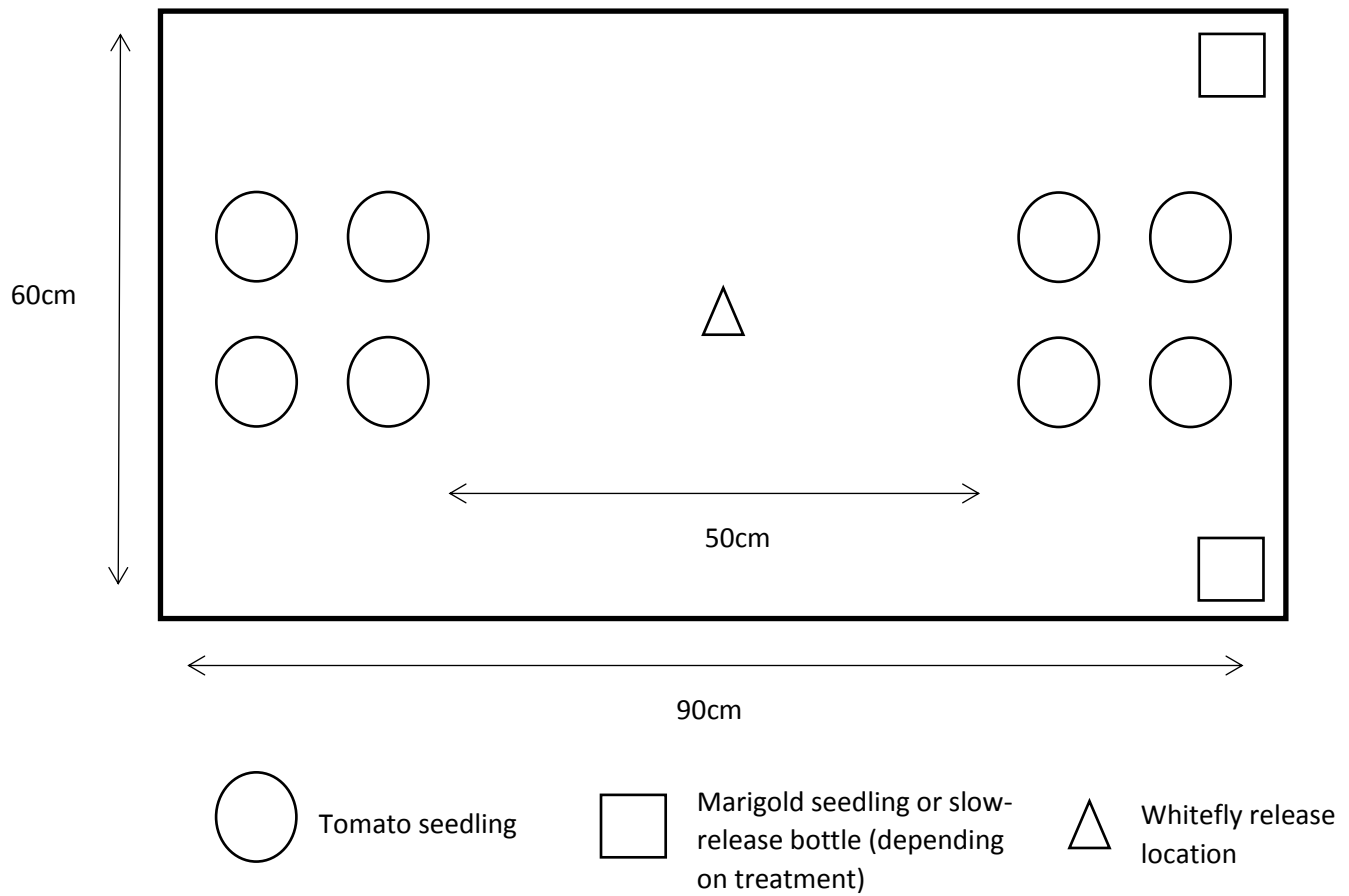

Supplement: S7 Fig — This diagram shows a plan view of the experimental design employed for the whitefly free-choice assays. (PDF) [file pone.0213071.s007.pdf]
