# Supplementary material for: Companion planting with French marigolds protects tomato plants from glasshouse whiteflies through the emission of airborne limonene
Source: PLoS One. 2019 Mar 1;14(3):e0213071. doi: 10.1371/journal.pone.0213071 (PMC6396911; doi:10.1371/journal.pone.0213071)
Supplement: S6 Fig — The non-choice plant tissue preference assay may be seen in S6A Fig, with 8 disks of tomato in each dish. Fifty whiteflies were added and the average number of whiteflies settled on plant tissue after 21h was calculated. Average whiteflies settled (n = 8) on each plant species can be seen in S6B Fig, with 95% confidence intervals for the mean plotted, and the species ordered in order of preference. This quantification of preference agreed with previous broad surveys of T. vaporariorum plant range (S6C Fig) from CABI [35], Roditakis [36] and Mound and Halsey [37], respectively, with non-hosts being less preferred and hosts more preferred. ‘+’ indicates ‘host’, ‘-’ indicates ‘non-host’ and ‘0’ indicates that this plant was not considered. Methods for this experiment can be found in S1 materials and methods. (PDF) [file pone.0213071.s006.pdf]

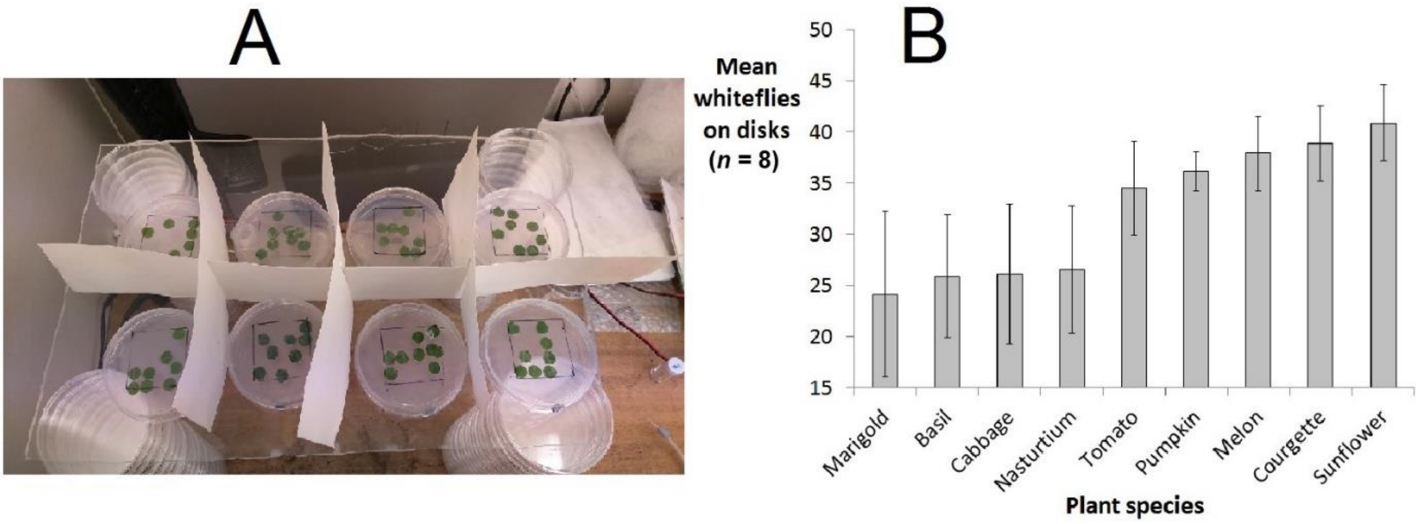

C

	M	B	C	N	**T**	P	Me	Co	S
	Marigold	Basil	Chinese cabbage	Nasturtium	**Tomato**	Pumpkin	Melon	Courgette	Sunflower
CABI, 2013	+	-	+	-	+	+	+	+	+
Roditakis, 1990	0	+	0	0	0	0	+	0	+
Mound and Halsey, 1978	-	-	-	+	+	+	+	+	+

Low → High: Whitefly plant preference
