# Supplementary material for: Companion planting with French marigolds protects tomato plants from glasshouse whiteflies through the emission of airborne limonene
Source: PLoS One. 2019 Mar 1;14(3):e0213071. doi: 10.1371/journal.pone.0213071 (PMC6396911; doi:10.1371/journal.pone.0213071)
Supplement: S8 Fig — Layout of experiments to test the efficacy of ‘push’ and ‘push-pull’ strategies against the glasshouse whitefly on tomato, and the efficacy of intercropping at reducing large whitefly population sizes. A randomised block design was used, with each of the 8 replicates containing all 3 treatments for each study in a random order. The ‘push’ experiment involved intercropping tomato with non-hosts. The ‘push-pull’ experiment was similar but additionally had attractive host plants around the perimeter. Whitefly plant preference for the various hosts was determined in laboratory leaf disk experiments (S6 Fig) and confirmed by literature surveys. The location of heavily infested aubergine plants used to supplement natural whitefly populations are shown with triangles containing the letter ‘W’. The experiment to test the introduction of plants during an advanced whitefly infestation was nearly identical in layout to the ‘Push’ experiment, but with limonene dispensers placed in compost replacing the non-tomato plant species in the HD treatment used the ‘push’ assay. Acronyms for the treatments used are as follows; C—control, LD—low diversity, HD—high diversity. For the individual plants within each treatment; T—tomato, M—marigold, N—nasturtium, B—Basil, C–Chinese cabbage, Me–Melon, Co–courgette, S–sunflower. (PDF) [file pone.0213071.s008.pdf]

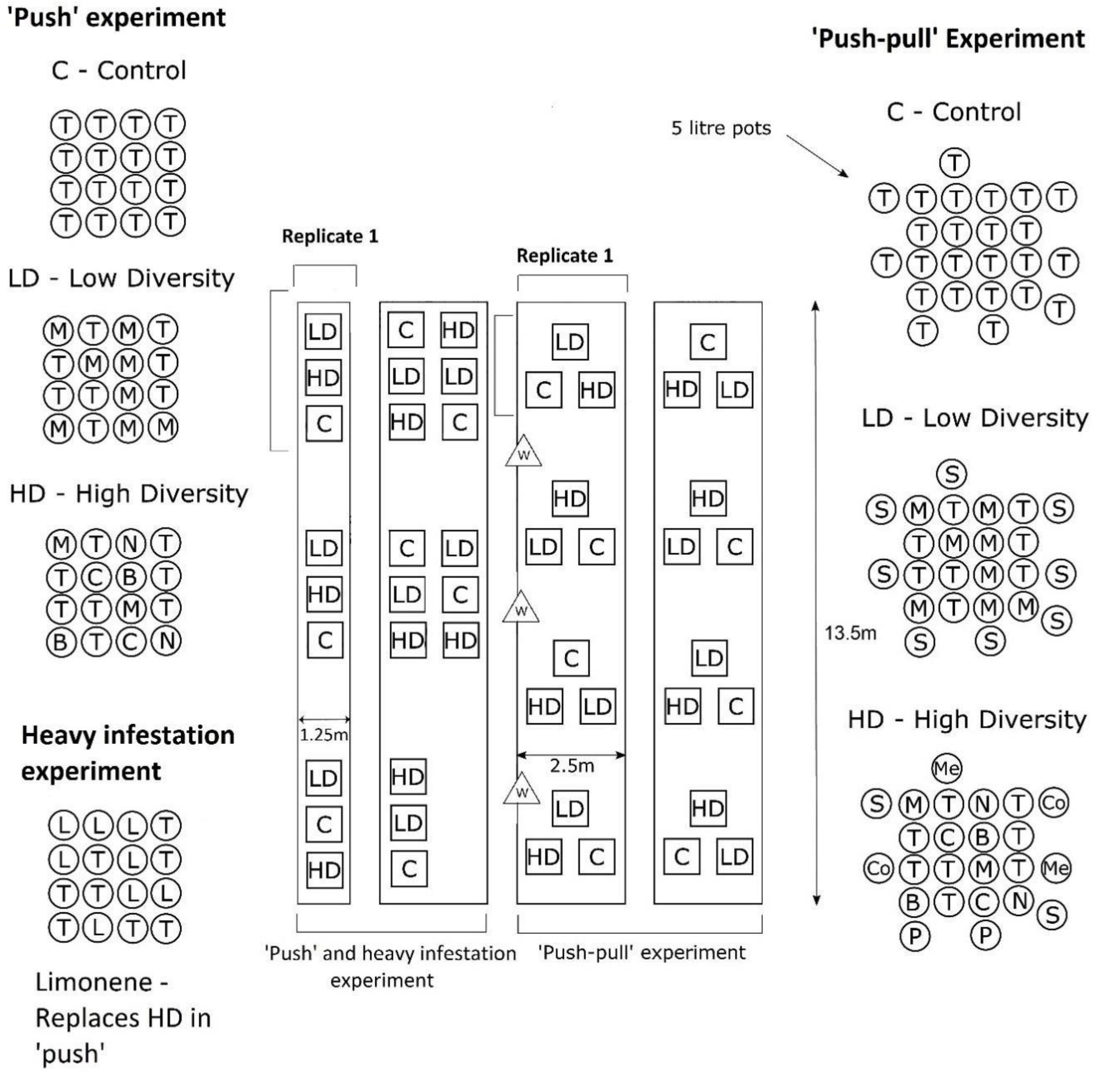
'Push' experiment
C - Control
T T T T
T T T T
T T T T
T T T T
LD - Low Diversity
M T M T
T M M T
T T M T
M T M M
HD - High Diversity
M T N T
T C B T
T T M T
B T C N
Heavy infestation experiment
L L L T
L T L T
T T L L
T L T T
Limonene - Replaces HD in 'push'
Replicate 1
Replicate 1
1.25m
2.5m
13.5m
W
'Push' and heavy infestation experiment
'Push-pull' experiment
'Push-pull' Experiment
5 litre pots
C - Control
LD - Low Diversity
HD - High Diversity
