# Supplementary material for: Companion planting with French marigolds protects tomato plants from glasshouse whiteflies through the emission of airborne limonene
Source: PLoS One. 2019 Mar 1;14(3):e0213071. doi: 10.1371/journal.pone.0213071 (PMC6396911; doi:10.1371/journal.pone.0213071)
Supplement: S1 Materials and Methods — (DOCX) [file pone.0213071.s011.docx]

**S1 Materials and Methods**

**Materials and Methods S5 Fig**

Whitefly response to marigold plants and limonene was tested using a 4-way olfactometer, this consisted of an enclosed Perspex arena of 30cm in diameter with four side wings, as described in [1]. This was chosen as it provides a constant flow of air which was at a rate of 0.2l/min through each wing. At the end of each of the four arms a ‘partial non-return’ glass bulb was fitted. The scent of the odour sources was added to the airflow by connecting four plastic airtight boxes to each wing of the olfactometer. For the control, a single “elegance” tomato leaflet from seedlings at stage 13 on the BBCH scale was placed in a 5ml vial filled with water inside each plastic box on each of the four wings of the olfactometer. One hundred whiteflies of mixed sex (obtained from the laboratory culture described in the main text materials and methods) were released into the centre of the olfactometer and were left for 24 hours, at which point the number of adult whiteflies in each of the four wings were recorded. For each of the four treatments (marigold flower, ML1, ML2 and limonene) one of the tomato leaflets in the olfactometer was replaced with a marigold flower, marigold leaves with a weight of approximately 1.7g (ML 1 treatment), marigold leaves with a weight of approximately 3.4g (ML2 treatment) and a “type 1” limonene dispenser (limonene treatment). The specific weights for ML1 and ML2 were based on the finding that marigold flowers (with an average weight of 1.7g) produce on average roughly twice the amount of limonene as marigold leaf tissue (see S4 Fig B). The entire apparatus was cleaned between each experiment and covered with an opaque sheet during experimental procedures to ensure the light intensity was approximately equal. The experiment was repeated four times per treatment. The Pearson’s chi-squared test was used to test if distribution of whiteflies differed significantly from the average settling distribution across 4 control replicates where only tomato was present in all four wings of the olfactometer.

**Materials and Methods S6 Fig**

To quantify whitefly preference for these plants, a laboratory leaf disk assay (shown in S6 Fig) was used to examine whitefly preference in microcosm, by monitoring how readily whiteflies colonised leaf disks of the different plant species in a no-choice situation and comparing these numbers to whitefly numbers on tomato leaf disks. This assay was modified from a previous study [2] and findings were compared to previous surveys of *T. vaporariorum* host range [3-5]. Plant species and varieties used in the laboratory assay and in glasshouse experiments were as follows: French marigold, *Tagetes patula* ‘honeycomb’; basil, *Ocimum basilicum* ‘sweet’; Chinese cabbage, *Brassica rapa* ‘Blues F1’; nasturtium, *Tropaeolum majus* ‘jewel mixed’; tomato, *Solanum lycopersicum* (plum) ‘Roma VF’; pumpkin, *Cucurbita pepo* ‘Racer F1’; melon, *Cucumis melo* ‘Antalya F1’; courgette, *Cucurbita pepo* (cylindrica) ‘All green bush’; sunflower, *Helianthus annuus* ‘Giant single’. For the leaf disk assays plants were grown in John Innes No. 2 compost in 9-cm-diameter and 8.7-cm-deep pots, at a density of one plant per pot, with plants watered liberally. All plants were grown under the same conditions as previously described in the “plants” section of the materials and methods of the main text. Plants had the following number of fully expanded leaves (not including the cotyledons) when used, which approximated to stage 13 on the BBCH scale for tomato, Chinese cabbage and sunflower: marigold 4-6 leaves, basil 2-4 leaves, Chinese cabbage 4-5 leaves, nasturtium 4-5 leaves, tomato 4 leaves, pumpkin 2-3 leaves, melon 3 leaves, courgette 1-2 leaves, and sunflower 4 leaves.

Sixteen 1cm diameter leaf disks were removed from each plant using a cork borer, with a total of 4 plants used per plant species. Hot 1% agar was poured into 90mm diameter 16mm height petri dishes to the point where it covered the bottom of the dish and 8 newly-cut leaf disks were pressed into the agar (once it had cooled and was on the point of setting) adaxial side down in random positions in a 50mm^2^ square in the centre of the dish. The lid was secured and the petri dish was then turned over. Fifty mixed sex adult whiteflies were removed from the laboratory culture described in the main text materials and methods and introduced into the dish through a side hole which was subsequently sealed. A 21h leaf disk assay *in situ* is shown in S6 Fig. Eight replicates of this design were completed for each plant species and were completed simultaneously, with 1 plant supplying 16 disks for 2 dishes so that 4 plants provided the disks for the full 8 replicates. Dishes were left for 21h at 20^o^C, 16h light / 8h dark, synchronized with cultures and plant propagation facilities, after which the total number of whiteflies settled on plant tissue within each dish was counted, with the mean whitefly colonisation number calculated from the 8 replicated dishes.

**References**

1. J. P. An Aphid Sex Attractant1970.

2. Frei A, Gu HN, Bueno JM, Cardona C, Dorn S. Antixenosis and antibiosis of common beans to Thrips palmi Karny (Thysanoptera : Thripidae). J Econ Entomol. 2003;96(5):1577-84. doi: Doi 10.1603/0022-0493-96.5.1577. PubMed PMID: WOS:000186236600027.

3. Trialeurodes vaporariorum [Internet]. 2013. Available from: <http://www.cabi.org/isc>.

4. Mound LA, Halsey SH. Whitefly of the World: A Systematic Catalogue of the Aleyrodidae (Homoptera) with Host Plant and Natural Enemy Data. : British Museum (Natural History); 1978.

5. Roditakis NE. Host plants of greenhouse whitefly Trialeurodes vaporariorum westwood (Homoptera: Aleyrodidae) in crete. Attractiveness and impact on whitefly life stages. Agriculture, Ecosystems & Environment. 1990;31(3):217-24. doi: <http://dx.doi.org/10.1016/0167-8809(90)90221-X>.
